# Current Therapeutic Options for the Main Monogenic Autoinflammatory Diseases and PFAPA Syndrome: Evidence-Based Approach and Proposal of a Practical Guide

**DOI:** 10.3389/fimmu.2020.00865

**Published:** 2020-06-03

**Authors:** Alessandra Soriano, Marco Soriano, Gerard Espinosa, Raffaele Manna, Giacomo Emmi, Luca Cantarini, José Hernández-Rodríguez

**Affiliations:** ^1^Division of Internal Medicine, Department of Internal Medicine and Medical Specialties, Arcispedale S. Maria Nuova – IRCCS, Reggio Emilia, Italy; ^2^School of Medicine, Luigi Vanvitelli University, Naples, Italy; ^3^Clinical Unit of Autoinflammatory Diseases and Vasculitis Research Unit, Department of Autoimmune Diseases, Hospital Clinic, IDIBAPS, University of Barcelona, Barcelona, Spain; ^4^Fondazione Policlinico Universitario A. Gemelli IRCCS and Periodic Fevers Research Centre, Institute of Internal Medicine, Università Cattolica Sacro Cuore, Rome, Italy; ^5^Department of Experimental and Clinical Medicine, University of Firenze, Firenze, Italy; ^6^Research Center of Systemic Autoinflammatory Diseases and Behçet's Disease, Rheumatology Unit of the Department of Medical Sciences, Surgery and Neurosciences, University of Siena, Siena, Italy

**Keywords:** monogenic autoinflammatory diseases, PFAPA syndrome, colchicine, anakinra, canakinumab, anti-TNF agents, tocilizumab, Janus kinase (JAK) inhibitors

## Abstract

Monogenic autoinflammatory diseases are rare conditions caused by genetic abnormalities affecting the innate immunity. Previous therapeutic strategies had been mainly based on results from retrospective studies and physicians' experience. However, during the last years, the significant improvement in their genetic and pathogenic knowledge has been accompanied by a remarkable progress in their management. The relatively recent identification of the inflammasome as the crucial pathogenic mechanism causing an aberrant production of interleukin 1β (IL-1β) in the most frequent monogenic autoinflammatory diseases led to the introduction of anti–IL-1 agents and other biologic drugs as part of the previously limited therapeutic armamentarium available. Advances in the treatment of autoinflammatory diseases have been favored by the use of new biologic agents and the performance of a notable number of randomized clinical trials exploring the efficacy and safety of these agents. Clinical trials have contributed to increase the level of evidence and provided more robust therapeutic recommendations. This review analyzes the treatment of the most frequent monogenic autoinflammatory diseases, namely, familial Mediterranean fever, tumor necrosis factor receptor–associated periodic fever syndrome, hyperimmunoglobulin D syndrome/mevalonate kinase deficiency, and cryopyrin-associated periodic syndromes, together with periodic fever with aphthous stomatitis, pharyngitis, and cervical adenitis syndrome, which is the most common polygenic autoinflammatory disease in children, also occurring in adult patients. Finally, based on the available expert consensus recommendations and the highest level of evidence of the published studies, a practical evidence-based guideline for the treatment of these autoinflammatory diseases is proposed.

## Introduction

Over the past 20 years, pathogenic insights into the mechanisms of autoinflammation completely changed treatment and prognosis of many inherited autoinflammatory diseases, also known as “hereditary periodic fever syndromes,” a group of rare diseases characterized by difficult diagnostic and therapeutic approaches. The most extensively studied and better pathogenically defined monogenic autoinflammatory conditions have been familial Mediterranean fever (FMF), tumor necrosis factor (TNF) receptor–associated periodic fever syndrome (TRAPS), hyperimmunoglobulin D syndrome/mevalonate kinase deficiency (HIDS/MKD), and cryopyrin-associated periodic syndromes (CAPS), which comprise three disorders with the same genetic background, and different phenotypes and outcomes. The CAPS spectrum includes familial cold autoinflammatory syndrome (FCAS), the mildest form; Muckle-Wells syndrome (MWS), the intermediate presentation; and chronic infantile neurological, cutaneous, and articular syndrome (CINCA) or neonatal-onset multisystem inflammatory disorder (NOMID), the most severe disease (1).

Until the late 1990s, traditional drugs, such as colchicine and glucocorticoids, had been used to treat autoinflammatory diseases. The pathogenic and therapeutic revolution started when NLRP3, one of the NOD-like receptors (NLRs) and part of the NLRP3 inflammasome, was discovered as the main actor in the activation of caspase 1 and the subsequent production of active interleukin 1β (IL-1β) (2). Mutations of genes involved in the inflammasome function or its related pathways were then identified as responsible for most of the monogenic autoinflammatory disorders recognized so far, also known as inflammasomopathies (3).

The discovery of the aberrant production of IL-1β as the final cause of all inflammasomopathies led to the introduction of anti–IL-1 agents and other biologic drugs to the very limited therapeutic armamentarium available for such diseases until then (4). In addition, the more recent knowledge of other autoinflammatory mechanisms, such as the activation of nuclear factor κB (NF-κB) and type I interferon (IFN) pathways, also provided new therapeutic options, such as anti-TNF and Janus kinase (JAK) inhibitors agents, directed to the specific blockade of cytokines and molecules involved in these novel inflammatory mechanisms (5–9).

Because monogenic autoinflammatory diseases are rare conditions, therapeutic strategies had been mainly based on results from retrospective studies and expert physicians' experience. Nevertheless, during the last years, the vast improvement in their genetic and pathogenic knowledge has been accompanied by a great effort in improving their management. In this sense, a remarkable number of randomized clinical trials exploring the efficacy and safety of new biologic agents in autoinflammatory diseases have been conducted, and therefore, therapeutic recommendations can be based now on higher evidence levels.

## Objectives and Methodology of the Review

This review intends to analyze the use of conventional and biologic agents in the most prevalent monogenic autoinflammatory diseases (FMF, TRAPS, HIDS/MKD, and CAPS) and periodic fever syndrome, aphthous stomatitis, pharyngitis, and cervical adenitis (PFAPA) syndrome. Although PFAPA syndrome is a polygenic or multifactorial disease with an unidentified genetic background, its treatment is reviewed because PFAPA syndrome is the most frequent autoinflammatory condition in childhood and has to be frequently considered part of the differential diagnosis of some monogenic disorders in children and adults (10).

Evidence-based recommendations for the management of FMF, TRAPS, HIDS/MKD, and CAPS have been provided from previous consensus studies using the European League Against Rheumatism (EULAR) standard operating procedures for developing best practice (Table 1) (11, 12), such as the EULAR recommendations for the management of FMF (13), and the European SHARE (Single Hub and Access point for pediatric Rheumatology in Europe) recommendations for the management of CAPS, TRAPS, and MKD (14). For all the autoinflammatory diseases, results of new clinical trials, international multicenter collaborative studies and registries, and retrospective data preferably from referral centers, have also been used to generate levels of evidence. The most relevant international multicenter initiatives and registries include the PRINTO Registry (a secured web-based registry hosted at the Pediatric Rheumatology International Trial Organization website - PRINTO, http://www.printo.it) and EUROFEVER Registry (an international registry for autoinflammatory diseases, https://www.printo.it/eurofever/) (15).

**Table 1:** Categorization of the level of clinical evidence and strength of recommendation based on the EULAR standardized operating procedures for EULAR-endorsed recommendations (11, 12).

**Category**	**Evidence from**
**LEVELS OF EVIDENCE**	
1A	Meta-analysis of randomized controlled trials
1B	At least one randomized controlled trial
2A	At least one controlled study without randomization
2B	At least one type of quasi-experimental study
3	Descriptive studies, such as comparative studies, correlation studies, or case–control studies
4	Expert committee reports or opinions and/or clinical experience of respected authorities
**Grade**	**Directly based on**
**STRENGTH OF RECOMMENDATION**	
A	Category 1 evidence
B	Category 2 evidence or extrapolated recommendation from category 2 evidence
C	Category 3 evidence or extrapolated recommendation from category 1 or 2 evidence
D	Category 4 evidence or extrapolated recommendation from category 2 or 3 evidence

Because, by definition, the strength of recommendation has to be based on a combination of data extracted from systematic literature review (providing levels of evidence) and consensus expert opinion (providing homogeneous and measurable information to generate the recommendations), in those conditions or situations for which any new therapeutic information has been reported but no opinion of the experts has been issued yet, only the level of evidence is mentioned (11, 12).

## Traditional Drugs

### Colchicine

Colchicine is an alkaloid extracted from two plants of the Lily family: *Colchicum autumnale* and *Gloriosa superba*. Historically, the therapeutic role of colchicine was for the first time raised up for the treatment of gout in the sixth century (16). More recently, colchicine use has been extended to other diseases such as primary biliary cirrhosis, Behçet disease, recurrent pericarditis, alcohol induced hepatitis, psoriasis, scleroderma, Sweet syndrome, amyloidosis, sarcoidosis and, in lesser extent, to inflammatory disorders prone to fibrosis (17–19).

In 1972, the efficacy of colchicine was first described in five FMF patients who experienced a dramatic reduction of the number of attacks (20). Since then, colchicine has become the basis of FMF treatment, and later on, several clinical trials definitively established its efficacy in preventing attacks and developing amyloidosis (21–23).

Colchicine was officially approved in 2009 by the US Food and Drug Administration (FDA) for the acute flares of gout and FMF, as a single-ingredient oral formulation (Colcrys^®^) (24). Nowadays, according to 2016 EULAR recommendations for the management of FMF, colchicine represents the first-line treatment in FMF, with a level of evidence 1B and grade of recommendation A (13).

Among other biological functions, colchicine has anti-inflammatory properties based on the inhibition of leukocyte chemotaxis, which is caused by its interaction with tubulin and the resulting dysfunction of microtubules. Microtubules, composed by α- and β-tubulin heterodimers, are involved in cell division, signal transduction, migration, secretion, and regulation of gene expression (25). Colchicine has the ability to bind in a poorly reversible manner to soluble non-polymerized tubulin in the cells, with the formation of a tubulin–colchicine complex (26, 27), and the subsequent movement inhibition of intracellular granules (28).

The predominant effect of colchicine on leukocytes, and more specific on granulocytes, has been correlated with the high concentrations of the drug in neutrophils compared to lymphocytes and monocytes. In this regard, defects in the efflux pumps with low activity of the P-glycoprotein 1 (PGY-1) efflux pump of granulocytes might explain the accumulation of colchicine in their cytoplasm (29, 30).

Other anti-inflammatory effects of colchicine include the reduction of TNF-α production by macrophages and TNF-α receptors on endothelial cells and the abrogation of neutrophil binding to adhesion molecules on vascular endothelium. Colchicine also decreases phospholipase A_2_ activity, release of lysosomal enzymes, and phagocytosis (31–33).

After the discovery of the *MEFV* gene as the gene encoding for pyrin and responsible for FMF, *MEFV* was found fully expressed in neutrophils, and some authors evidenced a colchicine-related cytosolic modulation of pyrin expression (34–36). Finally, colchicine has been shown to suppress the activation of caspase 1, the enzymatic component of NLRP3 inflammasome, with the subsequent inability to convert pro–IL-1β to active IL-1β (37).

Pharmacokinetic properties of colchicine include a narrow therapeutic range because the half-life after oral ingestion ranges between 7 and 9 h. The drug is absorbed in the jejunum and ileum and is metabolized in the liver by the cytochrome P (CYP) 450 3A4 and PGY-1. It is finally excreted mainly by the biliary, intestinal, and renal systems. Colchicine use in pregnant or nursing patients is considered relatively safe as long as hepatic and renal functions are intact. In this regard, because colchicine is metabolized via CYP3A4, its concomitant administration with agents that inhibit this isoenzyme (e.g., macrolide antibiotics, azole antifungals, statins) may produce elevated colchicine plasma concentrations, resulting in severe, and sometimes fatal complications. Colchicine intoxication may be also induced in patients with renal and/or liver disorders (38, 39).

#### Colchicine in Autoinflammatory Diseases

##### Familial Mediterranean Fever

Therapeutic doses of colchicine to treat FMF should be adjusted to the body weight, with a mean optimal dose of 0.03 ± 0.02 mg/kg per day. Total doses usually range from 0.5 to 2 mg/d in children and from 1 to 3 mg/d in adults (40, 41). Colchicine usually reduces severity, duration, and frequency of the attacks in the majority of FMF patients. Among them, ~30–40% of individuals experience a partial response (42). After ruling out any colchicine-associated gastrointestinal intolerance, other adverse effect, or poor patient compliance, only 5–10% of FMF cases can be finally considered colchicine-resistant FMF (crFMF) patients (15, 43).

Intravenous colchicine has shown efficacy in some patients unresponsive or with partial response to the oral formulation (43–45). An adjunctive weekly infusion of (1 mg) colchicine in FMF patients with frequent attacks despite a maximum tolerated oral dosage of 2–3 mg/d was associated with 50% reduction in frequency of attacks after 6 months (45). However, because of the potential risk of toxicity, intravenous colchicine is currently not recommended in FMF patients unresponsive to the oral formulation.

##### Other Autoinflammatory Diseases

Colchicine has not demonstrated effectiveness in HIDS/MKD and CAPS (14, 15). However, it has shown to be somehow effective in TRAPS and PFAPA syndrome. In the Eurofever Registry, colchicine was used in 39 TRAPS patients, with complete and partial response in 3 (8%) and 18 (46%) individuals, respectively (15). In a recent study of 24 patients with TRAPS, colchicine resulted to be of some help in 71% of cases, with a complete response in 12.5% of them. No differences in colchicine response were observed with regard to age at disease onset (pediatric vs. adult), type of variant (low vs. high penetrance), and colchine daily doses (1 mg vs. higher doses) (46). In 303 PFAPA patients from a tertiary Turkish center, colchicine was used as regular prophylactic treatment with high rate of response in terms of reduction of the frequency of episodes. Interestingly, heterozygous mutations in the *MEFV* gene were found in 25% of PFAPA subjects, who obtained even better results in terms of reduction of attacks. The potential modifier role of *MEFV* mutations in PFAPA patients seems associated with attenuated disease severity and higher response rate to colchicine compared to non-carriers of *MEFV* variants (47–49).

### Non-steroidal Anti-inflammatory Drugs

The main therapeutic effects of non-steroidal anti-inflammatory drugs (NSAIDs) are determined by the inhibition of cyclooxygenases, enzymes that convert arachidonic acid in prostaglandins, and thromboxanes. Some of these eicosanoids participate in different pathways involved in the inflammatory response. However, because NSAIDs do not inhibit the biosynthetic cascade originating arachidonic acid, these drugs do not usually influence the underlying cause of the disease.

#### NSAIDs in Autoinflammatory Diseases

NSAIDs have been used in monogenic autoinflammatory diseases as symptomatic treatment, alone or in addition to the baseline therapy. Although a complete response has been reported in a minority of patients with autoinflammatory conditions in the Eurofever Registry (8% of FMF and TRAPS, 13% of HIDS/MKD, 6% of CAPS, and 4% of PFAPA patients), NSAIDs, alone or in combination with glucocorticoids, appear to be of some help in 70–80% of all cases (15).

In some FMF patients with exertional leg pain and protracted febrile myalgia, similarly to glucocorticoids, NSAIDs seem to be also effective (50). Protracted febrile myalgia is a condition characterized by prolonged episodes of muscle pain affecting limbs with marked systemic inflammatory response in the absence of rhabdomyolysis. The EULAR recommendations for treating this complication with NSAIDs have a level of evidence 2B and grade of recommendation C (13).

Patients with TRAPS associated with R92Q mutations have been reported to respond better to the combination of colchicine and NSAIDs than those carrying clearly pathogenic *TNFRSF1A* mutations (15, 51). However, in TRAPS, HIDS/MKD, and CAPS patients, the use of NSAIDs as pain relievers during inflammatory attacks is recommended with a level of evidence 3 and strength of recommendation D (for TRAPS) and C (for HIDS/MKD, CAPS, and PFAPA syndrome) (14, 15).

### Glucocorticoids

Glucocorticoids suppress the production and effects of several proinflammatory mediators, induce the inhibition of leukocytes migration to the inflammatory foci, and interfere with the function of fibroblasts and endothelial cells, thereby exerting a powerful anti-inflammatory action, both on acute manifestations (i.e., pain, edema) and on late inflammatory stages (including reparative processes, such as cell proliferation and fibrosis). Glucocorticoids bind to a specific intracytoplasmic receptor, a ligand-activated transcription factor with both positive and negative effects on the regulation of gene expression. Consequently, the activated glucocorticoid receptor–glucocorticoid complex up-regulates the expression of anti-inflammatory proteins and suppresses the transcription of proinflammatory cytokines and chemokines by blocking the NF-κB signaling pathway (52).

#### Glucocorticoids in Autoinflammatory Diseases

##### Familial Mediterranean Fever

In FMF patients, when colchicine becomes insufficient to control the disease activity, glucocorticoids (always in combination with colchicine) have shown positive effects with variable response in 83% of the 47 cases recruited from the Eurofever Registry (15). The use of intravenous methylprednisolone (40 mg) on demand has also demonstrated efficacy in decreasing fever and abdominal and pleuritic pain during attacks in a study of 31 FMF patients (53).

FMF patients with protracted febrile myalgia may be controlled with intravenous and/or oral administration of high-dose glucocorticoids (54). In fact, glucocorticoids have been recommended for treating this complication with a level of evidence 2B and grade of recommendation C (13). A recent study on eight FMF patients with protracted febrile myalgia has shown good response with the use of intravenous methylprednisolone pulses, at a dose of 10 mg/kg per day, for 3 days, followed by oral glucocorticoids, at initial doses of 1–2 mg/kg and gradual tapering down over 6 weeks (55). A small comparative study on 15 FMF patients with protracted febrile myalgia suggested similar efficacy of oral glucocorticoids and NSAIDs (50).

##### Tumor Necrosis Factor Receptor–Associated Periodic Fever Syndrome

In TRAPS patients, prednisone (or equivalent) may be useful on demand, administered during the attacks at a dose of 1 mg/kg per day, usually with rapid tapering and cessation in the following days (51, 56, 57). In the Eurofever Registry, short-term glucocorticoids, with or without concomitant NSAIDs, were considered effective for controlling inflammatory attacks in 91% of TRAPS patients, with complete response in 41% of them (15). Glucocorticoids do not seem to have any significant effect in preventing amyloidosis, and its intermittent use does not reduce either the frequency of attacks or subclinical inflammation between them. Glucocorticoids in TRAPS are recommended either as short-term courses or as maintenance treatment with a level of evidence 3 and strength of recommendation C (14).

As in other monogenic autoinflammatory disorders, some TRAPS patients may require long-term glucocorticoid therapy. In these glucocorticoid-dependent situations, a biological agent is often warranted in order to avoid steroid side effects. Indeed, almost 80% of TRAPS patients initially treated with glucocorticoids may finally receive biologic agents as maintenance therapy to control symptoms (14, 58).

##### Hyperimmunoglobulin D Syndrome/Mevalonate Kinase Deficiency

Similarly to TRAPS patients, in HIDS/MKD, high-dose glucocorticoids may be useful during the attacks with some benefit in 73% of cases. However, complete improvement is achieved by only 9% of patients, and glucocorticoids do not decrease neither the intensity nor the frequency of the acute episodes (15). Short-term glucocorticoids, with or without NSAIDs, may be effective for alleviating inflammatory attacks and are recommended with a level of evidence 3 and strength of recommendation C (14).

##### Cryopyrin-Associated Periodic Syndromes

In CAPS patients, glucocorticoids, mostly used on demand, resulted in some benefit in 80% of patients included in the Eurofever Registry. However, glucocorticoids do not control the underlying inflammatory process or decrease the frequency of the attacks (15, 59). Anyway, for symptomatic adjunctive therapy, short courses of NSAIDs and glucocorticoids may be used, with a level of evidence 3 and strength of recommendation C (14). However, they should not be used for primary maintenance therapy (level of evidence 4 and grade of recommendation D) (14).

##### PFAPA Syndrome

Glucocorticoids are the treatment of choice in PFAPA syndrome, because their prompt administration is able to discontinue the attacks rapidly and completely in the majority of patients. Such dramatic response is usually considered a peculiar diagnostic feature in pediatric and adult patients (15, 60–63). The conventional dosage from 0.5 to 2 mg/kg of prednisone (or equivalent) in a single dose at the time of fever onset has been proved useful in a randomized clinical trial (64), and therefore, glucocorticoids can be recommended in PFAPA patients with a level of evidence 2B (15). However, additional doses of glucocorticoids may be occasionally necessary. Their use does not prevent further attacks and may even be associated with an increased frequency of the attacks in 25–50% of cases (15, 60).

Of note, the evidence for the effectiveness of tonsillectomy in children with PFAPA syndrome is based on a systematic review that included two small randomized controlled trials studying the effects of tonsillectomy compared to no surgery. Tonsillectomy was associated with immediate and complete clinical resolution and significant reduction in the frequency and severity of symptoms (65). Although these results have to be interpreted considering the clinical severity, the previous response to a single dose of prednisone, and the surgical risk in every individual situation, tonsillectomy as a therapy for PFAPA syndrome can be recommended with a level of evidence 1A (15).

### Other Conventional Agents

#### Thalidomide

Thalidomide exerts its immunomodulatory and anti-inflammatory actions through inhibiting TNF-α and IFN-γ synthesis, leukocyte chemotaxis, and angiogenesis. Teratogenicity and polyneuropathy are known as the most feared thalidomide-related serious adverse events. Thalidomide is an accepted treatment for dermatological diseases (such as leprous erythema nodosum, severe mucosal ulcers, cutaneous lupus erythematosus, and chronic graft-vs.-host disease), refractory multiple myeloma, and other systemic autoimmune or inflammatory conditions. Evidence on the efficacy of thalidomide in autoinflammatory diseases is limited to sporadic case reports and series. In three crFMF patients treated with thalidomide in addition to colchicine, the combination did not show clear benefit (66). Similarly, thalidomide failed to demonstrate any biological or clinical effect in six patients with HIDS/MKD (67).

#### Dapsone

Dapsone is an antibacterial sulfonamide drug with anti-inflammatory properties due to the inhibition of function and chemotaxis of neutrophils and blockade of the inflammatory effects of multiple prostaglandins and leukotrienes. Dapsone is currently used for the treatment of different infectious and immune-mediated systemic and dermatologic conditions (68). In monogenic autoinflammatory diseases, dapsone has been used in a case series of 10 crFMF patients with control of attacks in half of them. The authors postulated that dapsone might be considered as an alternative therapy in selected FMF cases not responding to colchicine (68).

#### Interferon α

The use of IFN-α in patients with FMF resistant to colchicine has been reported with controversial results. While in several crFMF cases IFN-α seemed to induce a marked decrease in both severity and frequency of the attacks, alone or in combination with colchicine (69–72), a double-blind randomized clinical trial of 22 FMF patients comparing IFN-α with placebo (without concomitant colchicine) did not demonstrate clear efficacy of IFN-α in reducing severity of attacks or inflammatory markers (73).

#### Other Drugs

Other anti-inflammatory, immune-modulatory, or immunosuppressive agents, such as azathioprine, methotrexate, cyclosporine, leflunomide, mycophenolate mofetil, sulfasalazine, statins, cimetidine, and antihistamines, have been historically used in monogenic autoinflammatory diseases mostly with unclear or unsatisfactory results (15, 51).

## Biologic Agents

### Interleukin 1 Blockers

It is well-known that IL-1 production has strong impact on initiation and maintenance of inflammatory process. Both IL-1α and IL-1β bind to the IL-1 receptor type, which is expressed by nearly every human cell and is responsible for triggering the cascade of inflammatory processes (74). IL-1 inhibitory molecule was first described over 30 years ago in the urine from patients with fever and monocytic leukemia (75, 76). This molecule was later cloned and identified as IL-1 receptor antagonist (IL-1Ra) (77), and subsequently, it was hypothesized that the inhibition of IL-1 with IL-1Ra could be a potential therapeutic option for treating inflammatory diseases (78).

With regard to inflammasome-mediated autoinflammatory diseases, such as FMF, TRAPS, HIDS/MKD, and CAPS, IL-1 blockade has become the most specific and useful treatment, as first-line therapy or when previous conventional anti-inflammatory or immunosuppressive agents are not useful. Although several IL-1 blockers are currently under development (79), the three biologic agents targeting IL-1 with special interest in autoinflammatory diseases, commercially available nowadays, are anakinra, canakinumab, and rilonacept.

Table 2 illustrates the most relevant studies supporting the maximum evidence level for the use of IL-1 blockers in the main monogenic autoinflammatory diseases (crFMF, TRAPS, HIDS/MKD, and CAPS) and PFAPA syndrome and includes the doses used in pediatric and adult patients.

**Table 2:** Anti-IL-1 agents, types of studies supporting the maximum evidence level for their use, and common pediatric and adult doses given in the main monogenic autoinflammatory diseases (crFMF, TRAPS, HIDS/MKD, and CAPS) and PFAPA syndrome.

**Anti-IL-1 agent**	**Disease**	**Type of study**	**EMA/FDA approval**	**Doses (children)**	**Doses (adults)**	**References**
Anakinra	crFMF	RCT, RCS	No/No	50–300 mg/day	50–100 mg/day	(80, 81)
	TRAPS	OLS, CS	No/No	1.5 mg/kg/day	100 mg/day	(82, 83)
	HIDS/MKD	OLS	No / No	1 mg/kg/day^*^	100 mg/day^*^	(84)
	CAPS	OLS, RCS, CS	Yes/Yes^**^	1–1.5 mg/kg/day, 1–2 mg/kg/day, 1.5–8 mg/kg/day	100 mg/day, 1–2 mg/kg/day, 1.5–8 mg/kg/day	(85–92)
	PFAPA	CS, CR	No/No	1 mg/kg/day^*^	100 mg/day^*^	(93, 94)
Canakinumab	crFMF	RCT, OLS	Yes/Yes	2 mg/Kg q4w or q8w	150–300 mg q4w or q8w	(95–97)
	TRAPS	RCT, OLS	Yes/Yes	2 mg/Kg q4w or q8w	150–300 mg q4w or q8w	(95, 98)
	HIDS/MKD	RCT, OLS	Yes/Yes	2 mg/Kg q4w or q8w	150–300 mg q4w or q8w	(95, 99)
	CAPS	RCT, OLS	Yes/Yes	2–10 mg/Kg q4w or q8w	150–300 mg q4w or q8w	(100–104)
	PFAPA	CR	No/No	2 mg/kg q8w	150 mg q8w	(105, 106)
Rilonacept	crFMF	RCT	No/No	2.2 mg/Kg qw	160 mg qw	(107)
	CAPS	RCT, OLS	No/No	4.4 mg/Kg followed by 2.2 mg/Kg qw	320 mg followed by 160 mg qw	(108, 109)

* *In some cases, on demand administration at the beginning of the febrile episode may also be used*.

** *Only for CINCA/NOMID cases*.

*CAPS, cryopyrin-associated periodic syndromes; CINCA/NOMID, chronic infantile neurological, cutaneous and articular syndrome/neonatal onset multisystem inflammatory disorder; CR, case report; CS, case series; crFMF, colchicine-resistant familial Mediterranean fever; EMA, European Medicines Agency; FDA, Food and Drug Administration; HIDS/MKD, hyperimmunoglobulin D and periodic fever syndrome/mevalonate kinase deficiency; OLS, open-label study; PFAPA, periodic fever with aphthous stomatitis, pharyngitis and cervical adenitis; RCS, retrospective cohort study; RCT, randomized controlled trial; TRAPS, TNF-receptor associated periodic fever syndrome*.

#### Anakinra

Anakinra is a recombinant non-glycosylated form of the human IL-1 receptor antagonist (rhIL-1Ra) that binds to IL-1 receptor type I (IL-1RI) and acts as competitive inhibitor with IL-1α and IL-1β, in a way that mimics the activity of endogenous IL-1Ra (110). Anakinra has been approved by the FDA and European Medicines Agency (EMA) for rheumatoid arthritis and by the EMA for the treatment of Still disease, including systemic juvenile idiopathic arthritis and adult-onset Still disease. In 2012, anakinra was approved for the treatment of CINCA/NOMID in the United States. In the European Union, anakinra was approved by the EMA for all types of CAPS in 2013.

The recommended initial dose of anakinra is 100 mg/d subcutaneously in adults and 0.5–2 mg/kg per day in children, who may require increasing dosage up to 5–8 mg/kg per day to maintain remission. The need of higher requirements of anakinra in pediatric patients might be related to pharmacokinetics of the drug whose effective steady-state concentration in young children seems to be higher than in adults (14, 111). Anakinra terminal half-life ranges from 4 to 6 h, and ~80% of the drug is excreted by renal clearance (112). Consequently, the mean plasma clearance of the drug strongly depends on normal kidney function, and anakinra removal from plasma is minimally feasible through dialysis (113).

An animal study showed that up to two-thirds of serum IL-1Ra are able to cross blood–brain barrier. On clinical grounds, anakinra administration has determined improvement of memory and cognitive functions in CAPS patients (85, 114).

Safety data on anakinra comes from rheumatoid arthritis randomized controlled trials and long-term observational studies, in which good safety profiles have been observed with no increase of opportunistic infections or malignancies (141, 142). Data from the British Society of Rheumatology Biologic Register reported higher rates of serious infections of the skin and respiratory tract in subjects with more severe disease and higher exposure to glucocorticoids (143). The increased susceptibility to respiratory infections in patients treated with anti–IL-1 might be explained by the fact that IL-1β seems to play a role in the resistance to *Streptococcus pneumoniae* infection (144). Although the most frequent adverse event by far of anakinra is the injection site skin reaction, which is observed in up to 70% of patients, this local reaction tends to decline over time without the need of treatment discontinuation (145). Anakinra use during pregnancy has not been associated with an increased rate of miscarriages or congenital malformations, neither in a registry of 40 patients (146) nor in small series of FMF and CAPS patients (147, 148).

#### Canakinumab

Canakinumab is a fully humanized IgG1 monoclonal antibody that acts specifically against IL-1β. In 2009, canakinumab received the FDA approval for CAPS treatment, specifically for FCAS and MWS, and EMA approval for children older than 2 years and adults with all forms of CAPS. More recently, in 2016, the FDA and EMA approved canakinumab for the treatment of crFMF, TRAPS, and MKD, based on the results of the phase 3 Canakinumab Pivotal Umbrella Study in Three Hereditary Periodic Fevers (CLUSTER) trial (95). Additional indications of canakinumab include systemic juvenile idiopathic arthritis (by the FDA) and Still disease (systemic juvenile idiopathic arthritis and adult-onset Still disease) and gouty arthritis (by the EMA).

Given its long half-life of 26 days, canakinumab administration is recommended subcutaneously at 2–4 mg/kg in children and at a minimal dose of 150 mg in adults, every 4–8 weeks in both age groups. Patients with more severe phenotypes may require dose escalation. For instance, patients with CINCA/NOMID (the most severe CAPS phenotype) may require up to 8 mg/kg dosage every 4 weeks to control symptoms (100, 101). Similarly, in crFMF, TRAPS, and MKD patients, the recommended starting dose is usually 150 mg or 2 mg/kg (in children with body weight between 7.5 and 49 kg) every 4 weeks, with the possibility to increase up to 300 mg every 4 weeks (or 4 mg/kg in children), which is more often required by MKD patients (95).

Its penetrance of the blood–brain barrier seems incomplete because canakinumab does not normalize intrathecal inflammatory markers, as demonstrated in two case series of CINCA/NOMID patients (149, 150). Renal function does not influence the pharmacokinetics of canakinumab (151). Data on pregnancy and breastfeeding are still scarce. However, seven of eight pregnancies of patients on canakinumab were reported uneventful, and no developmental abnormalities were reported in four breastfed infants while mothers were on canakinumab treatment (152).

Mild infections, involving mostly the urinary tract and upper airway, represent the most frequent canakinumab-associated adverse events. However, serious infections have been reported in 5.4/100 patients-years in 285 patients included in the long-term CAPS registry (153) and in 7.4/100 patients-years of FMF, TRAPS, and MKD patients treated with higher doses of canakinumab in the CLUSTER trial (95).

Additional data on safety can be extrapolated from the randomized, double-blind, placebo controlled trial of canakinumab in patients with atherosclerotic disease, with more than 10,000 individuals and ~30% of them receiving placebo (154). Neutropenia and thrombocytopenia were observed in the treatment group, with more deaths attributed to infections or sepsis, however, with no statistically significant differences in the overall rates of adverse events compared with placebo (154).

#### Rilonacept

Rilonacept is a dimeric fusion glycoprotein consisting of the Fc portion of human IgG1 and the human IL-1 receptor extracellular domains that binds IL-1α and IL-1β. In 2008, rilonacept was the first agent approved by the FDA for the treatment of FCAS and MWS in patients older than 12 years, based on two sequential phase III clinical studies, which demonstrated rilonacept safety and effectiveness in adult patients with CAPS (108). Rilonacept obtained a marketing authorization only in the United States.

Initial dose in adults is recommended at 320 mg subcutaneously, followed by a weekly dose of 160 mg. In children older than 11 years and adolescents, the dose has to be adjusted at 4.4 mg/kg (maximum of 320 mg) and then 2.2 mg/kg (maximum of 160 mg) once weekly. The circulating half-life may vary from 6 to 9 days (155, 156). Being a large molecule, it is expected not to cross the blood–brain barrier and to be cleared primarily by the reticuloendothelial system rather than by the kidney. Therefore, a dose adjustment in patients with renal disease is not required (157).

Injection site reactions, headache, and upper respiratory tract and urinary infections have been described as the most common adverse events in CAPS patients treated with rilonacept (109). Data from studies and trials on gout and gouty arthritis suggest also that several laboratory changes may occur, including transient neutropenia and a small increase in liver transaminases, triglycerides, and creatine phosphokinase (158).

#### IL-1 Blockers in Autoinflammatory Diseases

##### Familial Mediterranean Fever

IL-1 blockade with anakinra is currently recommended for FMF in case of true colchicine resistance, with a level of evidence 2B and strength of recommendation B, and as an optional treatment for protracted febrile myalgia, with a level of agreement 2B and grade of recommendation C (13).

Although in 2011 the Eurofever Registry included only three FMF patients treated with anakinra with complete response (15), a 2013 literature review identified 30 FMF cases resistant or intolerant to colchicine treated with anakinra, and four with canakinumab, with good clinical and laboratory outcomes (110). A 2015 systematic review reported 64 patients treated with anakinra and 40 with canakinumab. A complete response without attacks occurred in 76.5 and 67.5% of patients on anakinra and canakinumab, respectively. In patients with established type AA amyloidosis, both anti–IL-1 agents demonstrated to reverse proteinuria (159). Anakinra has been administered on demand with efficacy to some selected crFMF patients, mainly those with prominent prodromal manifestations or recognizable triggers of the attacks (160).

Ben-Zvi et al. (80) enrolled 25 adult patients with crFMF in the first double-blind randomized placebo-controlled trial, aiming to assess efficacy and safety of anakinra at a dose of 100 mg/d during 4 months of treatment. Adult FMF patients experiencing at least one attack per month despite the maximum tolerated dose of colchicine (up to 3 mg/d) were enrolled. Patients treated with anakinra had an attack rate of 1.7 per month, whereas those receiving placebo suffered 3.5 attacks per month. In addition, anakinra was associated with better quality of life, and no differences in the development of adverse effects were found. Interestingly, the best results with anakinra were observed in terms of amelioration of joint manifestations (80). In a Turkish multicenter retrospective study of 172 crFMF patients, in whom 151 were treated with anakinra and 21 with canakinumab, both drugs reduced the yearly attack frequency from 16.8 to 2.4 (*p* < 0.001). Forty-two percent of crFMF patients were attack-free, and proteinuria was significantly reduced in those patients with amyloidosis (81).

Canakinumab has proved effective in several retrospective FMF reviews (161–163) and two open-label phase II studies with nine and seven crFMF patients (96, 97). Both phase II trials established the efficacy of canakinumab in reducing the frequency of FMF attacks and maintaining low levels of acute phase reactants, with no unexpected adverse events (96, 97). The recent FDA and EMA approval of canakinumab for patients with FMF resistant or intolerant to colchicine has been based on data from the CLUSTER trial (95).

A total of 63 patients with crFMF were randomized to receive canakinumab 150 mg or placebo every 4 weeks. At week 16, canakinumab compared with placebo produced a significantly higher response rate by day 15 (61 vs. 6%), higher rates of physician global assessment of disease activity (minimal/none) (65 vs. 9%), and higher C-reactive protein (CRP) levels ≤10 mg/L (68 vs. 6%) and serum amyloid A (SAA) levels ≤10 mg/L (65 vs. 9%) (95). Canakinumab also demonstrated a rapid and sustained disease control assessed with the Autoinflammatory Disease Activity Index (AIDAI) over 16 weeks, and approximately half of crFMF patients had inactive disease after the same period (164). In the open-label phase from weeks 16–40, all FMF patients with a previous complete response to canakinumab 150 mg every 4 weeks maintained the absence of flares with canakinumab 150 mg every 8 weeks. After that period, the same dose interval of every 8 weeks was sufficient to maintain disease control in 46% of patients. An increase in the dose to 300 mg every 4 weeks was required by 10% of crFMF patients (95).

Adverse events and serious adverse events were more frequent in canakinumab-treated patients than in those receiving placebo. Overall, the most frequently reported adverse events were infections (mostly those affecting the respiratory tract), headache, abdominal pain, and injection-site reactions. Rates of infections and serious infections were of 173.3 and 6.6 per 100 patient-years of treatment, respectively (95).

With regard to rilonacept, this agent was administered to 14 FMF patients with one or more attacks per month in a randomized double-blind, single-participant alternating treatment study, with a significant reduction in the number of FMF attacks. Injection site reactions were the only adverse events associated to rilonacept administration (107).

##### Tumor Necrosis Factor Receptor–Associated Periodic Fever Syndrome

Anti–IL-1 agents have demonstrated efficacy in the majority of TRAPS patients. Anakinra seems to be superior to etanercept in retrospective TRAPS studies (15). In the Eurofever Registry, anakinra provided some benefit in ~90% of TRAPS patients, with a complete remission in 67% of them (15). Anakinra has shown to be effective, both in continuous and on-demand administration (14, 15, 57, 82, 83, 165), and is recommended in TRAPS patients with a level of evidence 2B and strength of recommendation B (14).

Canakinumab induced a complete response in 19 of 20 TRAPS patients in an open-label, phase II study (98), but it has been recently labeled by the FDA and EMA for TRAPS patients based on data from the CLUSTER trial (95). In such trial, among the 46 TRAPS patients randomized to receive canakinumab 150 mg or placebo every 4 weeks, at week 16, canakinumab, compared to placebo, was associated with a higher response rate by day 15 (45 vs. 8%), higher rates of (minimal/none) physician global assessment of disease activity (45 vs. 4%), and higher CRP levels ≤10 mg/L (36 vs. 8%) and SAA levels ≤10 mg/L (27 vs. 0%) at week 16. Canakinumab also produced a rapid and sustained disease control assessed with AIDAI over 16 weeks, and about half of TRAPS patients had inactive disease after the same period (164). In the open-label phase from week 16–40, 83% of TRAPS patients with a previous complete response to canakinumab 150 mg every 4 weeks maintained the absence of flares with canakinumab 150 mg every 8 weeks. After that period, the same dose interval of every 8 weeks was sufficient to maintain disease control in 53% of patients. An increase in the dose to 300 mg every 4 weeks was required by 8% of TRAPS patients (95).

Similarly to FMF patients of the CLUSTER study, TRAPS patients treated with canakinumab developed more frequently adverse events and serious adverse events than patients receiving placebo. The rate of infections was of 148 per 100 patient-years of treatment, and no serious infections were observed (95).

##### Hyperimmunoglobulin D Syndrome/Mevalonate Kinase Deficiency

In HIDS/MKD, anakinra, and canakinumab may control or attenuate the intensity of attacks in most of patients. In the Eurofever Registry, among the 62 HIDS/MKD patients treated with anakinra, 84% obtained a positive response, which was complete in only 29% of them (15). On-demand administration of anakinra in HIDS/MKD patients decreases the duration and severity of symptoms attacks when started within 24 h after the onset of symptoms. However, on-demand regimen does not influence the frequency of attacks (84). Anakinra is recommended for treating HIDS/MKD patients with a level of evidence 2B and strength of recommendation C (14).

Efficacy and safety of canakinumab have been recently investigated in an open-label phase II study in nine patients (six pediatric and three adults) with HIDS, using a predefined dosage of 300 mg or 4 mg/kg for patients ≤40 kg (higher than the usual dose of 150 mg used in CAPS), with an interval administration of every 6 weeks (99). A significant reduction of attacks frequency and complete clinical response with normalization of inflammatory markers within the first month of treatment were noted in all patients (99).

As for crFMF and TRAPS, canakinumab has also been recently approved by the FDA and EMA for HIDS/MKD treatment, based on the data derived from the CLUSTER trial (95). Among the 72 HIDS/MKD patients included in the study (treated with canakinumab 150 mg or placebo every 4 weeks), those receiving canakinumab experienced better response rate by day 15 (35 vs. 6%), higher rates of (minimal/none) physician global assessment of disease activity (46 vs. 6%), and higher CRP levels ≤10 mg/L (41 vs. 6%) and SAA levels ≤10 mg/L (41 vs. 6%) at week 16. Canakinumab was also associated with a rapid and sustained disease control assessed with AIDAI over 16 weeks, and 40% of HIDS/MKD patients had inactive disease after the same period (164). In the open-label phase from week 16–40, 82% of HIDS/MKD patients with a previous complete response to canakinumab 150 mg every 4 weeks maintained the absence of flares with canakinumab 150 mg every 8 weeks (95). After that period, the same dose interval of every 8 weeks maintained the disease controlled in only 23% of patients. An increase in the dose to 300 mg every 4 weeks was required by 29% of patients (95).

Similarly to FMF and TRAPS patients, in HIDS/MKD patients of the CLUSTER study, adverse events and serious adverse events were more frequent in patients treated with canakinumab than with placebo. Rates of infections and serious infections in HIDS/MKD were of 313.5 and 13.7 per 100 patient-years of treatment, which were higher than for FMF and TRAPS patients (95).

##### Cryopyrin-Associated Periodic Syndromes

Anakinra, canakinumab, and rilonacept are approved by the FDA and EMA for CAPS treatment. All the three anti–IL-1 agents are currently recommended as first-line therapy in CAPS patients of any age, with a level of evidence 1B for canakinumab and rilonacept, and 2A for anakinra, and a strength of recommendation A and B, respectively (14).

Anakinra has demonstrated to control clinical and biological activity in CAPS. FDA approval of anakinra was based on a long-term, open-label, and uncontrolled study of 18 CINCA/NOMID patients, in whom symptoms and inflammatory markers improved in all of them (86). This study provided relevant information about dosage variability of anakinra to control CAPS activity. Patients were treated with an initial dose of 1–2.4 mg/kg body weight and an average maintenance dose of 3–4 mg/kg per day (with a maximum dose administered of 7.6 mg/kg per day). Although most of individuals received a single daily dose, some of them achieved better control of disease by splitting the dose into two daily administrations. Upon withdrawal of treatment, disease flare occurred after a median time of 5 days (86).

Several open-label and prospective studies (85–90) and retrospective series (91, 92) supported EMA approval of anakinra in adults and pediatric patients 8 months or older with a body weight of 10 kg or greater, diagnosed with any type of CAPS [CINCA/NOMID (85, 86, 91, 92), MWS (85, 87, 88), and FCAS (88–90)].

Although anakinra does not seem to control the progression of osteoarticular deformities in CINCA/NOMID patients (166, 167), it improves leptomeningeal and cochlear involvement, due to its ability to penetrate the blood–brain barrier (15, 168, 169). Therefore, anakinra appears to be more effective than canakinumab in the intrathecal compartment (149).

Anakinra efficacy and safety were analyzed in a prospective, open-label, single-center clinical cohort study, including 43 severe CAPS patients followed during a mean of 5 years (170). Anakinra was safe and well-tolerated both in pediatric and adult patients, with most adverse events emerging during the first months after treatment initiation. The most frequent adverse events included headache, arthralgia, and injection site reactions. Infections, such as pneumonia and gastroenteritis, occurred in ~25% of patients, but they did not require permanent discontinuation of treatment. Interestingly, an increase of anakinra dose was required in two cases during an infectious event with a concomitant disease flare (170).

Canakinumab induced clinical and biological remission in 75–90% of CAPS patients in the Eurofever Registry (15). The approval of canakinumab for CAPS was based on a 48-week, double-blind, placebo-controlled trial of 35 patients with CAPS (mainly MWS patients) treated with 150 mg every 8 weeks, in which 97% of patients had a complete response to canakinumab during the study period (102). In addition, another randomized controlled trial (103) and two observational studies on CAPS patients including all disease phenotypes similarly showed good results (100, 104).

A prospective study comparing the efficacy and safety of canakinumab and anakinra in 26 MWS patients concluded that both agents equally controlled disease activity and inflammatory markers and that canakinumab may be effective in some patients not responding to anakinra (104). Occasionally, patients receiving anakinra and those treated with canakinumab (mainly CINCA/NOMID patients) may require an increase of dose or frequency of administration of the drug (101, 104).

Rilonacept showed efficacy in 47 adult patients with CAPS (44 with FCAS and 3 with MWS) in a randomized controlled trial and in an extended open-label study of up to 96 weeks including 101 patients with favorable safety and tolerability profile in adult and pediatric patients (108, 109).

##### PFAPA Syndrome

Although genetic and pathogenic mechanisms of PFAPA syndrome are unknown, in a case report and a small cohort of five PFAPA patients resistant to glucocorticoids, the administration of anakinra has been effective in suppressing disease flares during the long-term follow-up (93, 94). Similarly, a pediatric case and an adult case of PFAPA syndrome have been reported to respond to canakinumab at a dose of 2 mg/kg and 150 mg every 8 weeks, respectively (105, 106).

### TNF Blockers

Anti-TNF agents, mainly etanercept (a dimeric human TNF receptor p75-Fc fusion protein), infliximab (a chimeric monoclonal antibody against TNF-α), and adalimumab (a fully human monoclonal antibody against TNF-α), have been used in different monogenic autoinflammatory inflammasomopathies, generally with poorer efficacy than IL-1 blockers. These biologic agents have been approved for several autoimmune/inflammatory conditions. Etanercept has FDA and EMA indications for rheumatoid arthritis, juvenile idiopathic arthritis, ankylosing spondylitis, psoriatic arthritis, and plaque psoriasis; infliximab was approved by the FDA and EMA for rheumatoid arthritis, ankylosing spondylitis, psoriatic arthritis, plaque psoriasis, Crohn disease, and ulcerative colitis, and adalimumab is indicated by the FDA and EMA for all the previous diseases (approved for etanercept and infliximab) plus non-infectious uveitis. In addition, the FDA approved adalimumab as therapy for hidradenitis suppurativa (acne inversa). None of the anti-TNF drugs have been approved by either the FDA or EMA for any of the monogenic autoinflammatory diseases.

#### TNF Blockers in Autoinflammatory Diseases

##### Familial Mediterranean Fever

In FMF, the experience with anti-TNF agents has been overall scarce and unclear. Two small series of 10 and 14 patients with crFMF and concomitant inflammatory conditions (chronic arthritis, ankylosing spondylitis, juvenile idiopathic arthritis, psoriasis, or Crohn disease) treated with all the three TNF blockers resulted in good control of FMF and the associated disease (115, 116). Retrospective data included in the Eurofever Registry showed complete and partial response with etanercept (in seven and nine cases, respectively), infliximab (seven with complete and eight with partial response), and adalimumab (three with complete and two with partial response) (15). Similarly to the previous series, in this registry, anti-TNF agents seemed also to exert more benefit in cases with predominant arthritis (15). In a multicenter international retrospective study including 27 FMF patients treated with biologic agents, two patients received adalimumab and two etanercept as first agent. While one patient experienced a complete response, the remaining three had to discontinue the drug due to lack of efficacy after a mean duration of therapy of 9.3 months (117). The level of evidence of the efficacy of anti-TNF agents in crFMF, especially in those with articular involvement, is 3, but no grade of recommendation has been provided (13).

##### Tumor Necrosis Factor Receptor–Associated Periodic Fever Syndrome

In TRAPS, etanercept is the anti-TNF agent with the best reported results, because it has shown to prevent or reduce the intensity of attacks and the dose of glucocorticoids previously controlling disease activity (15, 117). However, it is relatively common that etanercept has to be discontinued because of lack of efficacy (15, 117), which has been reported after a period of 3.3 years (118). Although SHARE consensus recommends etanercept in some patients with a level of evidence 2B and grade of recommendation C, the experts also inform that the effect might decline over time (14).

In the Eurofever Registry, among 121 TRAPS patients treated with etanercept, 88% experienced a satisfactory response, which was complete in 26% of them (15). A multicenter international retrospective study including 47 TRAPS patients, 41 with pathogenic mutations and 6 with the R92Q variant, analyzed 20 and 4 patients treated with etanercept in each group, respectively. Among TRAPS caused by pathogenic mutations, ~50% of those receiving etanercept could achieve a complete clinical response. In addition, a total of 13 of 20 (65%) TRAPS patients discontinued anti-TNF, mostly due to a lack of efficacy. Compared to patients receiving anakinra, those treated with etanercept experienced less frequently a complete response and higher drug discontinuation rates (117). With regard to the four R92Q-TRAPS patients receiving etanercept as first-line therapy, only one (25%) had a complete response (117). Etanercept has also been reported effective in isolated TRAPS patients carrying the R92Q variant (57).

The use of infliximab and adalimumab has been associated with severe paradoxical reactions, and therefore, they are currently not recommended in TRAPS. While etanercept is a receptor fusion protein, infliximab, and adalimumab are TNF monoclonal antibodies. One of the proposed mechanisms by which infliximab is involved in a hyperinflammatory response is related to the failure in shedding infliximab-bound receptor from the cell surface, with the subsequent activation of antiapoptotic mechanisms and a widespread inflammatory response (171).

##### Hyperimmunoglobulin D Syndrome/Mevalonate Kinase Deficiency

In HIDS/MKD, anti-TNF therapy can improve frequency and intensity of attacks. However, because anti–IL-1 agents can be considered the first-line therapy for HIDS/MKD patients (14, 95), TNF blockers are recommended as a second option (together with IL-6 blockade) in case of IL-1 blockers are ineffective or not tolerated, with a level of evidence 4 and grade of recommendation D (14).

In the Eurofever Registry, etanercept was the most frequently used anti-TNF drug, providing any improvement in 26 of 44 (59%) of HIDS/MKD patients, which was complete in seven (16%) of them (15). A multicenter retrospective study of eight HIDS/MKD patients treated with etanercept showed higher complete response rates (88%). However, etanercept was discontinued in four patients (50%) due to lack of efficacy (117).

##### Cryopyrin-Associated Periodic Syndromes

No evidence of efficacy of biological therapy (including anti-TNF agents) other than IL-1 blockade in CAPS patients is available (119).

##### PFAPA Syndrome

To date, no PFAPA cases treated with anti-TNF agents have been reported.

### Anti–IL-6 Agents: Tocilizumab

Tocilizumab is a humanized monoclonal anti–IL-6 receptor antibody currently labeled by the FDA and EMA for rheumatoid arthritis, systemic and polyarticular juvenile idiopathic arthritis, giant cell arteritis and for the treatment of chimeric antigen receptor T cell–induced severe or life-threatening cytokine release syndrome. Tocilizumab has not been approved for any of the monogenic autoinflammatory diseases because the experience of its use, usually in patients unresponsive to other biologic agents, is still occasional.

#### Tocilizumab in Autoinflammatory Diseases

##### Familial Mediterranean Fever

Tocilizumab has been successful in controlling disease activity in the majority of patients with crFMF reported as single cases, with good control of secondary amyloidosis in some of them (120–125). Tocilizumab effects over proteinuria in FMF-associated amyloidosis has been analyzed in two series of 11 and 12 patients resistant to colchicine, anti–IL-1, or anti-TNF agents, in whom the previous colchicine therapy was maintained (126, 127). Proteinuria improvement in any degree was achieved by 7 of 11 patients (64%) and 9 of 12 patients (75%), respectively (126, 127). In two of the responder patients, tocilizumab was discontinued, and proteinuria returned, with good control after restarting IL-6 blockade (128).

##### Other Autoinflammatory Diseases

Tocilizumab has been successfully used in three patients with TRAPS (129–131) and in seven HIDS/MKD cases (132–136) in whom anti-TNF and/or anti–IL-1 agents had previously failed. Negative results have been reported in two CAPS (CINCA/NOMID) patients treated with tocilizumab (119, 137). No evidence on the use of tocilizumab in PFAPA syndrome is currently reported.

### JAK Inhibitors

JAK inhibition suppresses the constitutive phosphorylation of the transcription factor STAT-1, which blocks the induction of IFN-stimulated genes and subsequently leads to the regulation of the uncontrolled IFN production, causing inflammatory manifestations. Janus kinase inhibitors, such as tofacitinib, baricitinib, and ruxolitinib, are currently approved by the FDA and EMA for rheumatoid arthritis, psoriatic arthritis, and ulcerative colitis (tofacitinib), rheumatoid arthritis (baricitinib), and myelofibrosis and polycythemia vera (ruxolitinib, also approved for steroid-refractory acute graft-vs.-host disease by the FDA).

JAK inhibitors have demonstrated positive effects in several monogenic autoinflammatory diseases mediated by type I IFN (also named interferonopathies), including chronic atypical neutrophilic dermatosis with lipodystrophy and elevated temperature/proteasome-associated autoinflammatory syndrome, STING-associated vasculopathy with onset in infancy, familial chilblain lupus, and Aicardi–Goutières syndrome (172–175).

Because the pathogenic mechanism of the diseases included in this review consists mainly in the abnormal function of the inflammasome, as expected, the use of JAK inhibitors in monogenic inflammasomopathies remains anecdotal and limited to six crFMF patients previously failing to IL-1, TNF, and IL-6 blockers. However, interestingly, these cases were successfully treated with tofacitinib (138–140).

Table 3 summarizes all the biological agents (other than IL-1 blockers) used in the main monogenic autoinflammatory diseases and PFAPA syndrome, and types of studies supporting the maximum evidence level for their use.

**Table 3:** Biological agents (other than IL-1 blockers), types of studies supporting the maximum evidence level, and response to treatment of the main monogenic autoinflammatory diseases (crFMF, TRAPS, HIDS/MKD, and CAPS)^*^.

**Drug**	**Disease**	**Type of study**	**Response**	**References**
Anti-TNF*EtanerceptInfliximabAdalimumab*	crFMF	RCS	Partial or complete response in few patients. Better efficacy in patients with articular involvement	(15, 115, 116)
	TRAPS^**^	RCS	Good response in 88% of patients, complete in 25–50% of them	(15, 117, 118)
	HIDS/MKD	RCS	Good response in 59–88% of patients, complete in 16% of them Lack of efficacy over time	(15, 117)
	CAPS	CR	No response	(119)
Anti- IL-6*Tocilizumab*	crFMF	RCS, CS, CR	Good response in patients resistant to colchicine, anti-IL-1, and anti-TNF agents	(120–128)
	TRAPS	CR	Good response in patients refractory to anti-TNF and anti-IL-1 agents	(129–131)
	HIDS/MKD	CR	Good response in patients refractory to anti-TNF and anti-IL-1 agents	(132–136)
	CAPS	CR	No response	(119, 137)
JAK-inhibitors*Tofacitinib*	crFMF	CR, CS	Good response in patients refractory to anti-TNF, anti-IL-1, and anti-IL-6 agents	(138–140)

* *No evidence about the use of these drugs in PFAPA syndrome*.

** *Infliximab and adalimumab are not recommended in TRAPS since their use has been associated with severe paradoxical reactions*.

*CAPS, cryopyrin-associated periodic syndromes; CR, case report; CS, case series; crFMF, colchicine-resistant familial Mediterranean fever; HIDS/MKD, hyperimmunoglobulin D and periodic fever syndrome/mevalonate kinase deficiency; IL, interleukin, JAK, Janus kinase; PFAPA, periodic fever with aphthous stomatitis, pharyngitis and cervical adenitis; RCS, retrospective cohort study; TNF, tumor necrosis factor; TRAPS, TNF-receptor associated periodic fever syndrome*.

## Proposal of a Practical Guide for Treating the Main Monogenic Autoinflammatory Diseases and PFAPA Syndrome

**Figure 1:**
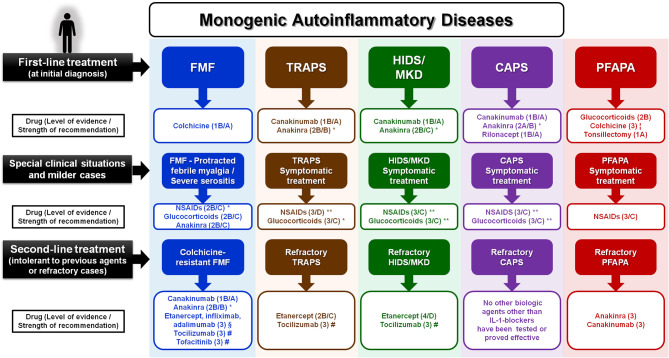
Proposed approach for the treatment of the main monogenic autoinflammatory diseases and PFAPA syndrome, based on the maximum level of evidence and grade of recommendation (when available) for each drug and disease. No strength of recommendation is provided when expert opinion consensus still does not exist. Thalidomide and dapsone have also been reported of benefit in some colchicine-resistant FMF patients (data within the text). *This drug has been proved effective with continuous and on demand administration. **This drug is recommended mostly in short courses (on demand) during attacks. ¦Colchicine in PFAPA patients has been reported more effective in carriers of heterozygous variants in the *MEFV* gene. §Anti-TNF agents (etanercept, infliximab, and adalimumab) have been shown more useful in FMF patients with prominent articular manifestations. #Tocilizumab has been used with efficacy in few patients with FMF, TRAPS, and HIDS/MKD who failed to anti–IL-1 and/or anti-TNF agents. #Tofacitinib has shown good response in few patients with colchicine-resistant FMF who also failed to IL-1, TNF, and IL-6 blockers. CAPS, cryopyrin-associated periodic syndrome; FMF, familial Mediterranean fever; HIDS/MKD, hyperimmunoglobulin D and periodic fever syndrome/mevalonate kinase deficiency; PFAPA, periodic fever with aphthous stomatitis, pharyngitis and cervical adenitis; TRAPS, TNF receptor–associated periodic syndrome.

Based on the maximum level of evidence and grade of recommendation for FMF, CAPS, TRAPS, HIDS/MKD, and PFAPA syndrome, a proposed practical scheme for the treatment of these conditions is illustrated in Figure 1. Evidence-based recommendations for the treatment of the main monogenic autoinflammatory diseases have been extracted from previous consensus studies, such as the EULAR recommendations for the management of FMF (13) and the European SHARE recommendations for the management of TRAPS, HIDS/MKD, and CAPS (14). Additional information used to generate levels of evidence has been incorporated mostly from results of new clinical trials, international multicenter registries, and retrospective data mainly from referral centers. According to the EULAR standard operating procedures for developing best practice to endorse recommendations, only the level of evidence is provided in those diseases with new therapeutic information but without recommendations issued by consensus expert opinion (11, 12).

The proposed therapeutic approach includes three different levels or situations: (a) first-line treatment (at the time of disease diagnosis or when symptomatic treatment used for milder situations is not effective); (b) milder cases or special clinical situations (including severe serositis or protracted febrile myalgia in case of FMF, or symptomatic treatment in milder presentations for the remaining conditions); and (c) second-line treatment (in case of patients not responding to first-line drugs or other biologic agents).

## Still Unsolved and Open Questions

Despite the great efforts and progresses achieved during the last years in the treatment of monogenic autoinflammatory diseases, several questions remain still pending to be answered:

- how to define resistance or failure to any biologic drug?
- how to demonstrate the effect of biologic agents in preventing secondary amyloidosis?
- which is the right therapeutic window to initiate or switch to a biologic agent?
- which biological agent should be used as first?
- when it is time to switch to another biologic agent, and which biologic?
- how to identify the right moment to start on-demand treatment and when changing from continuous to on demand administration is appropriate?
- how to establish, if possible, reduction and discontinuation of any treatment used?

## New Treatment Strategies in Autoinflammatory Diseases

It is of crucial importance to maintain and boost the international collaboration with patients' registries for all the autoinflammatory diseases and also to promote initiatives that allow the generation of genetic, clinical, and therapeutic research. The latter should include drug development in preclinical (phases I and II) trials, whereas phase III trials (evaluating safety) should be now focused on testing new drugs and also on performing head-to-head comparisons between the current biologic agents, respectively.

With regard to preclinical trials, because the abnormal activation of the NLRP3 inflammasome seems to be implicated in conditions other than autoinflammatory diseases, such as diabetes, atherosclerosis, and cardiovascular and neurodegenerative diseases, several *in vitro* and *in vivo* studies are exploring the potential role of different pharmacological inhibitors of the NLRP3 inflammasome in NLRP3-associated diseases (176). While some of these new agents are small molecule inhibitors directly acting on the NLRP3 protein, others are targeting different components and products of the inflammasome. These new agents include glyburide, 16673-34-0, JC124, FC11A-2, parthenolide, VX-740, VX-765, Bay 11-7082, BHB, MNS, CY-09, OLT1177, oridonin, MCC950, and tranilast (176). Among them, tranilast and other small molecules, such as IZD174 and IZD334 (related to MCC950), are being used in patients with CAPS, in phases I and II clinical trials.

Currently, the efficacy and safety of alternative biologic drugs and small molecules are being studied or pending of results in several ongoing clinical trials, including:

1. Tocilizumab for the treatment of FMF—a randomized, double-blind, phase II proof-of-concept study (ClinicalTrials.gov identifier: NCT03446209). This trial will analyze the efficacy of tocilizumab in adult patients with crFMF, also failing to other biologic agents.
2. A randomized, double-blind, parallel-group comparison trial of tocilizumab for colchicine-resistant FMF (UMIN-CTR Clinical Trial ID: UMIN000028010). This (phase III) trial will analyze the efficacy of tocilizumab in patients from 12 to 75 years with crFMF (177).
3. An open-label continuation trial of tocilizumab for FMF with colchicine ineffective or intolerance (UMIN-CTR Clinical Trial ID: UMIN000032557). This trial is expected to obtain evidence regarding the long-term safety of tocilizumab in crFMF patients (178).
4. Safety and tolerability, pharmacokinetic, and pharmacodynamic study with IZD334 (ClinicalTrials.gov identifier: NCT04086602). This is the first-in-human, single-center, double-blind, randomized, crossover (phase I) study of IZD334 (a small molecule inhibitor of the NLRP3 inflammasome) conducted in healthy adults as well as an open-label cohort in adult patients with CAPS.
5. Safety and tolerability, pharmacokinetic, and pharmacodynamic study with inzomelid (ClinicalTrials.gov identifier: NCT04015076). A phase I, randomized, double-blind, placebo-controlled, single- and multiple-ascending-dose study to determine the safety, tolerability, pharmacokinetics, pharmacodynamics, and food effect of inzomelid (or IZD174, a small molecule inhibitor of the NLRP3 inflammasome) in healthy adult participants, as well as an open-label cohort to confirm the safety, pharmacokinetics, and pharmacodynamics of inzomelid in adult patients with CAPS.
6. A clinical study of tranilast in the treatment of CAPS (ClinicalTrials.gov identifier: NCT03923140). A single-arm prospective cohort (phase II) study designed to observe the efficacy and safety of tranilast (a small molecule inhibitor of the NLRP3 inflammasome) in CAPS patients.
7. Ilaris^®^ (canakinumab) in PFAPA patients (ClinicalTrials.gov identifier: NCT02775994). This single-arm open-label pilot study will analyze the efficacy of canakinumab during the first 2 months of treatment in 10 children with PFAPA syndrome experiencing frequent flares.

## Concluding Remarks

- Colchicine, used at therapeutic doses, is the criterion-standard treatment for FMF and of some help in PFAPA patients, mostly in those carrying heterozygous *MEFV* gene mutations.
- NSAIDs improve symptoms in most patients with any autoinflammatory disease, but they are usually insufficient to control symptoms and do not influence the underlying cause of the disease in any of them.
- Glucocorticoids, generally used at medium or high doses, may be effective when administered on demand (in short courses) in most of the monogenic conditions. In some TRAPS patients, continuous administration of glucocorticoids may be also useful. Anyway, because most patients will require a biologic agent to control the disease activity, glucocorticoids may be reserved as initial treatment to prove response or may be also administered in a continuous or on-demand modality for mild/non-severe cases. Glucocorticoids have demonstrated special utility in FMF patients experiencing protracted febrile myalgia and severe serositis (in a short course at high doses) and in PFAPA patients (in a single high-dose administration), representing the treatment of choice in PFAPA syndrome.
- Among biologic agents, IL-1 blockers are the most relevant and useful drugs in the treatment of the main monogenic autoinflammatory diseases, especially in those considered inflammasomopathies.
- Anakinra and canakinumab have FDA and EMA approval as first-line treatment for CAPS patients, whereas rilonacept has been approved only by the FDA. Both anakinra and canakinumab are of clear utility in crFMF, TRAPS, and HIDS/MKD patients, and canakinumab has been recently approved by the FDA and EMA for these three conditions. Anakinra has been used with success on demand in patients with all monogenic diseases and PFAPA syndrome and therefore may be reserved for selected patients, mainly for those presenting with prominent manifestations or recognizable triggers preceding the attacks.
- Information regarding other alternative biologic agents is scarce and extracted from small case series and case reports including patients who did not respond to other biologics.
- Anti-TNF agents, and in particular, etanercept, seem to exert some benefit in FMF patients with prominent articular manifestations and also in TRAPS and HIDS/MKD patients.
- Tocilizumab (anti–IL-6 agent) has been successful in controlling disease activity and improving proteinuria in patients with FMF-associated amyloidosis and also in few TRAPS and HIDS/MKD patients resistant to anti-TNF and/or anti–IL-1 agents.
- The use of JAK inhibitors in monogenic inflammasomopathies is limited to the use of tofacitinib with good response in few crFMF patients previously failing to IL-1, TNF, and IL-6 blockers.
- The knowledge of genetic and pathogenesis of these autoinflammatory diseases has to be encouraged in order to discover new potential molecular targets leading to new specific drugs.
- Further randomized clinical trials proving the efficacy and safety of the currently promising biologic drugs and the new potential agents and small molecules are still needed.

## Author Contributions

AS and JH-R designed, wrote and reviewed the article, and created the original figure. MS, GEs, RM, GEm, and LC collaborated with the writing and the revision of the manuscript.

## Conflict of Interest

The authors declare that the research was conducted in the absence of any commercial or financial relationships that could be construed as a potential conflict of interest.

## References

[1] Federici S Sormani MP Ozen S Lachmann HJ Amaryan G Woo P. Evidence-based provisional clinical classification criteria for autoinflammatory periodic fevers. Ann Rheum Dis. (2015) 74:799–805. 10.1136/annrheumdis-2014-206580 25637003

[2] Martinon F Burns K Tschopp J. The inflammasome: a molecular platform triggering activation of inflammatory caspases and processing of proIL-beta. Mol Cell. (2002) 10:417–26. 10.1016/S1097-2765(02)00599-3 12191486

[3] Soriano A Pras E. Familial mediterranean fever: genetic update. Isr Med Assoc J. (2014) 16:274–6. 24979829

[4] Ozen S Bilginer Y. A clinical guide to autoinflammatory diseases: familial Mediterranean fever and next-of-kin. Nat Rev Rheumatol. (2013) 10:135–47. 10.1038/nrrheum.2013.174 24247370

[5] Aksentijevich I Zhou Q. NF-kB pathway in autoinflammatory diseases: dysregulation of protein modifications by ubiquitin defines a new category of autoinflammatory diseases. Front Immunol. (2017) 8:399. 10.3389/fimmu.2017.00399 28469620 PMC5395695

[6] de Jesus AA Goldbach-Mansky R. Newly recognized mendelian disorders with rheumatic manifestations. Curr Opin Rheumatol. (2015) 27:511–9. 10.1097/BOR.0000000000000207 26196376 PMC4565793

[7] Lee-Kirsch MA. The type i interferonopathies. Annu Rev Med. (2017) 68:297–315. 10.1146/annurev-med-050715-104506 27813875

[8] Sun SC. Non-canonical NF-kappaB signaling pathway. Cell Res. (2011) 21:71–85. 10.1038/cr.2010.177 21173796 PMC3193406

[9] Manthiram K Zhou Q Aksentijevich I Kastner DL The monogenic autoinflammatory diseases define new pathways in human innate immunity and inflammation. Nat Immunol. (2017) 18:832–42. 10.1038/ni.3777 28722725

[10] Cattalini M Soliani M Lopalco G Rigante D Cantarini L. Systemic and organ involvement in monogenic autoinflammatory disorders: a global review filtered through internists' lens. Intern Emerg Med. (2016) 11:781–91. 10.1007/s11739-016-1466-y 27221072

[11] Dougados M Betteridge N Burmester GR Euller-Ziegler L Guillemin F Hirvonen J. EULAR standardised operating procedures for the elaboration, evaluation, dissemination, and implementation of recommendations endorsed by the EULAR standing committees. Ann Rheum Dis. (2004) 63:1172–6. 10.1136/ard.2004.023697 15308532 PMC1755117

[12] van der Heijde D Aletaha D Carmona L Edwards CJ Kvien TK Kouloumas M. Update of the EULAR standardised operating procedures for EULAR-endorsed recommendations. Ann Rheum Dis. (2015) 74:8–13. 10.1136/annrheumdis-2014-206350 25261577 PMC4283681

[13] Ozen S Demirkaya E Erer B Livneh A Ben-Chetrit E Giancane G. EULAR recommendations for the management of familial mediterranean fever. Ann Rheum Dis. (2016) 75:644–51. 10.1136/annrheumdis-2015-208690 26802180

[14] Ter Haar NM Oswald M Jeyaratnam J Anton J Barron KS Brogan PA Recommendations for the management of autoinflammatory diseases. Ann Rheum Dis. (2015) 74:1636–44. 10.1136/annrheumdis-2015-207546 26109736

[15] Ter Haar N Lachmann H Ozen S Woo P Uziel Y Modesto C. Treatment of autoinflammatory diseases: results from the eurofever registry and a literature review. Ann Rheum Dis. (2013) 72:678–85. 10.1136/annrheumdis-2011-201268 22753383

[16] Malkinson FD. Colchicine. new uses of an old, old drug. Arch Dermatol. (1982) 118:453–7. 10.1001/archderm.118.7.453 7046640

[17] Ben-Chetrit E Levy M. Familial mediterranean fever. Lancet. (1998) 351:659–64. 10.1016/S0140-6736(97)09408-7 9500348

[18] Ben-Chetrit E Levy M. Colchicine: 1998 update. Semin Arthritis Rheum. (1998) 28:48–59. 10.1016/S0049-0172(98)80028-0 9726336

[19] Cocco G Chu DC Pandolfi S. Colchicine in clinical medicine. a guide for internists. Eur J Intern Med. (2010) 21:503–8. 10.1016/j.ejim.2010.09.010 21111934

[20] Goldfinger SE. Colchicine for familial mediterranean fever. N Engl J Med. (1972) 287:1302. 10.1056/NEJM197212212872514 4636899

[21] Dinarello CA Wolff SM Goldfinger SE Dale DC Alling DW. Colchicine therapy for familial mediterranean fever. a double-blind trial. N Engl J Med. (1974) 291:934–7. 10.1056/NEJM197410312911804 4606353

[22] Zemer D Revach M Pras M Modan B Schor S Sohar E. A controlled trial of colchicine in preventing attacks of familial mediterranean fever. N Engl J Med. (1974) 291:932–4. 10.1056/NEJM197410312911803 4606109

[23] Zemer D Pras M Sohar E Modan M Cabili S Gafni J. Colchicine in the prevention and treatment of the amyloidosis of familial mediterranean fever. N Engl J Med. (1986) 314:1001–5. 10.1056/NEJM198604173141601 3515182

[24] FDA Alert Information for Healthcare Professionals: New Safety Information for Colchicine (marketed as Colcrys). (2009). Available online at: https://wwwfdagov/drugs/drugsafety/postmarketdrugsafetyinformationforpatientsandproviders/ucm174382htm.

[25] Kershenobich D Rojkind M Quiroga A Alcocer-Varela J. Effect of colchicine on lymphocyte and monocyte function and its relation to fibroblast proliferation in primary biliary cirrhosis. Hepatology. (1990) 11:205–9. 10.1002/hep.1840110208 2137807

[26] Sackett DL Varma JK. Molecular mechanism of colchicine action: induced local unfolding of beta-tubulin. Biochemistry. (1993) 32:13560–5. 10.1021/bi00212a023 8257691

[27] Vandecandelaere A Martin SR Engelborghs Y. Response of microtubules to the addition of colchicine and tubulin-colchicine: evaluation of models for the interaction of drugs with microtubules. Biochem J. (1997) 323:189–96. 10.1042/bj3230189 9173881 PMC1218294

[28] Andreu JM Timasheff SN. Interaction of tubulin with single ring analogues of colchicine. Biochemistry. (1982) 21:534–43. 10.1021/bi00532a019 7066305

[29] Klimecki WT Futscher BW Grogan TM Dalton WS. P-glycoprotein expression and function in circulating blood cells from normal volunteers. Blood. (1994) 83:2451–8. 10.1182/blood.V83.9.2451.bloodjournal8392451 7513198

[30] Ben-Chetrit E Levy M. Does the lack of the P-glycoprotein efflux pump in neutrophils explain the efficacy of colchicine in familial mediterranean fever and other inflammatory diseases? Med Hypotheses. (1998) 51:377–80. 10.1016/S0306-9877(98)90031-7 9848464

[31] Ding AH Porteu F Sanchez E Nathan CF. Downregulation of tumor necrosis factor receptors on macrophages and endothelial cells by microtubule depolymerizing agents. J Exp Med. (1990) 171:715–27. 10.1084/jem.171.3.715 2155279 PMC2187771

[32] Cronstein BN Molad Y Reibman J Balakhane E Levin RI Weissmann G. Colchicine alters the quantitative and qualitative display of selectins on endothelial cells and neutrophils. J Clin Invest. (1995) 96:994–1002. 10.1172/JCI118147 7543498 PMC185287

[33] Paya M Terencio MC Ferrandiz ML Alcaraz MJ. Involvement of secretory phospholipase A2 activity in the zymosan rat air pouch model of inflammation. Br J Pharmacol. (1996) 117:1773–9. 10.1111/j.1476-5381.1996.tb15353.x 8732290 PMC1909548

[34] Mansfield E Chae JJ Komarow HD Brotz TM Frucht DM Aksentijevich I. The familial Mediterranean fever protein, pyrin, associates with microtubules and colocalizes with actin filaments. Blood. (2001) 98:851–9. 10.1182/blood.V98.3.851 11468188

[35] Abedat S Urieli-Shoval S Shapira E Calko S Ben-Chetrit E Matzner Y. Effect of colchicine and cytokines on MEFV expression and C5a inhibitor activity in human primary fibroblast cultures. Isr Med Assoc J. (2002) 4:7–12. 11802319

[36] Ben-Chetrit E Bergmann S Sood R. Mechanism of the anti-inflammatory effect of colchicine in rheumatic diseases: a possible new outlook through microarray analysis. Rheumatology. (2006) 45:274–82. 10.1093/rheumatology/kei140 16188942

[37] Martinon F Petrilli V Mayor A Tardivel A Tschopp J. Gout-associated uric acid crystals activate the NALP3 inflammasome. Nature. (2006) 440:237–41. 10.1038/nature04516 16407889

[38] Rochdi M Sabouraud A Girre C Venet R Scherrmann JM. Pharmacokinetics and absolute bioavailability of colchicine after i.v. and oral administration in healthy human volunteers and elderly subjects. Eur J Clin Pharmacol. (1994) 46:351–4. 10.1007/BF00194404 7957521

[39] Niel E Scherrmann JM. Colchicine today. Joint Bone Spine. (2006) 73:672–8. 10.1016/j.jbspin.2006.03.006 17067838

[40] Hentgen V Grateau G Kone-Paut I Livneh A Padeh S Rozenbaum M. Evidence-based recommendations for the practical management of familial mediterranean Fever. Semin Arthritis Rheum. (2013) 43:387–91. 10.1016/j.semarthrit.2013.04.011 23742958

[41] Ozkaya N Yalcinkaya F. Colchicine treatment in children with familial Mediterranean fever. Clin Rheumatol. (2003) 22:314–7. 10.1007/s10067-003-0739-9 14579163

[42] Soriano A Manna R. Familial mediterranean fever: new phenotypes. Autoimmun Rev. (2012) 12:31–7. 10.1016/j.autrev.2012.07.019 22878273

[43] Ozen S Kone-Paut I Gul A. Colchicine resistance and intolerance in familial mediterranean fever: definition, causes, and alternative treatments. Semin Arthritis Rheum. (2017) 47:115–20. 10.1016/j.semarthrit.2017.03.006 28413100

[44] Lidar M Kedem R Langevitz P Pras M Livneh A. Intravenous colchicine for treatment of patients with familial mediterranean fever unresponsive to oral colchicine. J Rheumatol. (2003) 30:2620–3. 14719203

[45] Rozenbaum M Boulman N Feld J Avshovich N Petrovich S Elias M. Intravenous colchicine treatment for six months: adjunctive therapy in familial mediterranean fever (FMF) unresponsive to oral colchicine. Clin Exp Rheumatol. (2009) 27:S105. 19796546

[46] Vitale A Sota J Obici L Ricco N Maggio MC Cattalini M Role of colchicine treatment in tumor necrosis factor receptor associated periodic syndrome (TRAPS): real life data from the AIDA Network. Mediators Inflamm. (2020). 10.1155/2020/1936960 PMC7273368 32565720

[47] Berkun Y Levy R Hurwitz A Meir-Harel M Lidar M Livneh A. The familial mediterranean fever gene as a modifier of periodic fever, aphthous stomatitis, pharyngitis, and adenopathy syndrome. Semin Arthritis Rheum. (2011) 40:467–72. 10.1016/j.semarthrit.2010.06.009 20828792

[48] Butbul Aviel Y Tatour S Gershoni Baruch R Brik R. Colchicine as a therapeutic option in periodic fever, aphthous stomatitis, pharyngitis, cervical adenitis (PFAPA) syndrome. Semin Arthritis Rheum. (2016) 45:471–4. 10.1016/j.semarthrit.2015.07.005 26315860

[49] Dusser P Hentgen V Neven B Kone-Paut I. Is colchicine an effective treatment in periodic fever, aphtous stomatitis, pharyngitis, cervical adenitis (PFAPA) syndrome? Joint Bone Spine. (2016) 83:406–11. 10.1016/j.jbspin.2015.08.017 27068612

[50] Kaplan E Mukamel M Barash J Brik R Padeh S Berkun Y Protracted febrile myalgia in children and young adults with familial mediterranean fever: analysis of 15 patients and suggested criteria for working diagnosis. Clin Exp Rheumatol. (2007) 25 (4 Suppl. 45):S114–7. 17949564

[51] Vitale A Rigante D Lucherini OM Caso F Muscari I Magnotti F. Biological treatments: new weapons in the management of monogenic autoinflammatory disorders. Mediators Inflamm. (2013) 2013:939847. 10.1155/2013/939847 23970817 PMC3736401

[52] Rhen T Cidlowski JA. Antiinflammatory action of glucocorticoids–new mechanisms for old drugs. N Engl J Med. (2005) 353:1711–23. 10.1056/NEJMra050541 16236742

[53] Erken E Ozer HT Bozkurt B Gunesacar R Erken EG Dinkci S. Early suppression of familial Mediterranean fever attacks by single medium dose methyl-prednisolone infusion. Joint Bone Spine. (2008) 75:370–2. 10.1016/j.jbspin.2007.10.004 18313967

[54] Langevitz P Zemer D Livneh A Shemer J Pras M. Protracted febrile myalgia in patients with familial mediterranean fever. J Rheumatol. (1994) 21:1708–9. 7799354

[55] Rom E Amarilyo G Levinski Y Bilavsky E Goldberg O Amir J. Protracted febrile myalgia syndrome treated with pulse of corticosteroids. Semin Arthritis Rheum. (2018) 47:897–9. 10.1016/j.semarthrit.2017.10.008 29129325

[56] Hernández-Rodríguez J Ruiz-Ortiz E Tomé A Espinosa G González-Roca E Mensa-Vilaró A. Clinical and genetic characterization of the autoinflammatory diseases diagnosed in an adult reference center. Autoimmun Rev. (2016) 15:9–15. 10.1016/j.autrev.2015.08.008 26299986

[57] Ruiz-Ortiz E Iglesias E Soriano A Buján-Rivas S Español-Rego M Castellanos-Moreira R. Disease phenotype and outcome depending on the age at disease onset in patients carrying the R92Q low-penetrance variant in TNFRSF1A gene. Front Immunol. (2017) 8:299. 10.3389/fimmu.2017.00299 28396659 PMC5366323

[58] Lachmann HJ Minden K Obici L Naselli A Insalaco A Hentgen V. Treatment responses in TRAPS: Eurofever/ Eurotraps. Pediatr Rheumatol Online J. (2013) 11(Suppl. 1):A188. 10.1186/1546-0096-11-S1-A18822377804

[59] Sag E Bilginer Y Ozen S. Autoinflammatory Diseases with Periodic Fevers. Curr Rheumatol Rep. (2017) 19:41. 10.1007/s11926-017-0670-8 28631068

[60] Feder HM Salazar JC. A clinical review of 105 patients with PFAPA (a periodic fever syndrome). Acta Paediatr. (2010) 99:178–84. 10.1111/j.1651-2227.2009.01554.x 19889105

[61] Cantarini L Vitale A Bartolomei B Galeazzi M Rigante D. Diagnosis of PFAPA syndrome applied to a cohort of 17 adults with unexplained recurrent fevers. Clin Exp Rheumatol. (2012) 30:269–71. 22325152

[62] Rigante D Vitale A Natale MF Lopalco G Andreozzi L Frediani B. A comprehensive comparison between pediatric and adult patients with periodic fever, aphthous stomatitis, pharyngitis, and cervical adenopathy (PFAPA) syndrome. Clin Rheumatol. (2017) 36:463–8. 10.1007/s10067-016-3317-7 27251674

[63] Hernández-Rodríguez J Ruiz-Ortiz E Yagüe J. Monogenic autoinflammatory diseases: General concepts and presentation in adult patients. Med Clin. (2018) 150:67–74. 10.1016/j.medcle.2017.11.043 28923673

[64] Yazgan H Gultekin E Yazicilar O Sagun OF Uzun L. Comparison of conventional and low dose steroid in the treatment of PFAPA syndrome: preliminary study. Int J Pediatr Otorhinolaryngol. (2012) 76:1588–90. 10.1016/j.ijporl.2012.07.020 22858452

[65] Burton MJ Pollard AJ Ramsden JD Chong LY Venekamp RP. Tonsillectomy for periodic fever, aphthous stomatitis, pharyngitis and cervical adenitis syndrome (PFAPA). Cochrane Database Syst Rev. (2019) 12:CD008669. 10.1002/14651858.CD008669.pub3 31886897 PMC6953364

[66] Seyahi E Ozdogan H Celik S Ugurlu S Yazici H. Treatment options in colchicine resistant familial mediterranean fever patients: thalidomide and etanercept as adjunctive agents. Clin Exp Rheumatol. (2006) 24:S99–103. 17067437

[67] Drenth JP Vonk AG Simon A Powell R van der Meer JW. Limited efficacy of thalidomide in the treatment of febrile attacks of the hyper-IgD and periodic fever syndrome: a randomized, double-blind, placebo-controlled trial. J Pharmacol Exp Ther. (2001) 298:1221–6. 11504824

[68] Salehzadeh F Jahangiri S Mohammadi E. Dapsone as an alternative therapy in children with familial mediterranean fever. Iran J Pediatr. (2012) 22:23–7. 23056855 PMC3448211

[69] Calguneri M Apras S Ozbalkan Z Ozturk MA Ertenli I Kiraz S. The efficacy of continuous interferon alpha administration as an adjunctive agent to colchicine-resistant familial Mediterranean fever patients. Clin Exp Rheumatol. (2004) 22:S41–4. 15515783

[70] Tankurt E Tunca M Akbaylar H Gonen O. Resolving familial Mediterranean fever attacks with interferon alpha. Br J Rheumatol. (1996) 35:1188–9. 10.1093/rheumatology/35.11.1188 8948315

[71] Tweezer-Zaks N Rabinovich E Lidar M Livneh A. Interferon-alpha as a treatment modality for colchicine- resistant familial Mediterranean fever. J Rheumatol. (2008) 35:1362–5. 18528960

[72] Vandecasteele SJ De Paepe P De Vriese AS. Successful treatment of renal AA amyloidosis in familial mediterranean fever with pegylated alpha-2a interferon. Clin Nephrol. (2011) 75(Suppl. 1):1–3. 10.5414/CN106447 21269584

[73] Tunca M Akar S Soyturk M Kirkali G Resmi H Akhunlar H. The effect of interferon alpha administration on acute attacks of familial mediterranean fever: a double-blind, placebo-controlled trial. Clin Exp Rheumatol. (2004) 22:S37–40. 15515782

[74] Dinarello CA Simon A van der Meer JW Treating inflammation by blocking interleukin-1 in a broad spectrum of diseases. Nat Rev Drug Discov. (2012) 11:633–52. 10.1038/nrd3800 22850787 PMC3644509

[75] Liao Z Grimshaw RS Rosenstreich DL. Identification of a specific interleukin 1 inhibitor in the urine of febrile patients. J Exp Med. (1984) 159:126–36. 10.1084/jem.159.1.126 6607312 PMC2187209

[76] Balavoine JF de Rochemonteix B Williamson K Seckinger P Cruchaud A Dayer JM. Prostaglandin E2 and collagenase production by fibroblasts and synovial cells is regulated by urine-derived human interleukin 1 and inhibitor(s). J Clin Invest. (1986) 78:1120–4. 10.1172/JCI112669 3020090 PMC423775

[77] Carter DB Deibel MR Jr Dunn CJ Tomich CS Laborde AL. Purification, cloning, expression and biological characterization of an interleukin-1 receptor antagonist protein. Nature. (1990) 344:633–8. 10.1038/344633a0 2139180

[78] Dinarello CA Thompson RC. Blocking IL-1: interleukin 1 receptor antagonist *in vivo* and *in vitro*. Immunol Today. (1991) 12:404–10. 10.1016/0167-5699(91)90142-G 1838480

[79] Stahl S Graslund T Eriksson Karlstrom A Frejd FY Nygren PA Lofblom J. Affibody molecules in biotechnological and medical applications. Trends Biotechnol. (2017) 35:691–712. 10.1016/j.tibtech.2017.04.007 28514998

[80] Ben-Zvi I Kukuy O Giat E Pras E Feld O Kivity S. Anakinra for colchicine-resistant familial mediterranean fever: a randomized, double-blind, placebo-controlled trial. Arthritis Rheumatol. (2017) 69:854–62. 10.1002/art.39995 27860460

[81] Akar S Cetin P Kalyoncu U Karadag O Sari I Cinar M. Nationwide experience with off-label use of interleukin-1 targeting treatment in familial mediterranean fever patients. Arthritis Care Res. (2018) 70:1090–4. 10.1002/acr.23446 28992387

[82] Obici L Meini A Cattalini M Chicca S Galliani M Donadei S Favourable and sustained response to anakinra in tumour necrosis factor receptor-associated periodic syndrome (TRAPS) with or without AA amyloidosis. Ann Rheum Dis. (2011) 70:1511–2. 10.1136/ard.2010.143438 21173015

[83] Gattorno M Pelagatti MA Meini A Obici L Barcellona R Federici S. Persistent efficacy of anakinra in patients with tumor necrosis factor receptor-associated periodic syndrome. Arthritis Rheum. (2008) 58:1516–20. 10.1002/art.23475 18438813

[84] Bodar EJ Kuijk LM Drenth JP van der Meer JW Simon A Frenkel J. On-demand anakinra treatment is effective in mevalonate kinase deficiency. Ann Rheum Dis. (2011) 70:2155–8. 10.1136/ard.2011.149922 21859689

[85] Lepore L Paloni G Caorsi R Alessio M Rigante D Ruperto N. Follow-up and quality of life of patients with cryopyrin-associated periodic syndromes treated with anakinra. J Pediatr. (2010) 157:310–5.e1. 10.1016/j.jpeds.2010.02.040 20472245

[86] Goldbach-Mansky R Dailey NJ Canna SW Gelabert A Jones J Rubin BI. Neonatal-onset multisystem inflammatory disease responsive to interleukin-1beta inhibition. N Engl J Med. (2006) 355:581–92. 10.1056/NEJMoa055137 16899778 PMC4178954

[87] Kuemmerle-Deschner JB Tyrrell PN Koetter I Wittkowski H Bialkowski A Tzaribachev N. Efficacy and safety of anakinra therapy in pediatric and adult patients with the autoinflammatory Muckle-Wells syndrome. Arthritis Rheum. (2011) 63:840–9. 10.1002/art.30149 21360513

[88] Hawkins PN Lachmann HJ Aganna E McDermott MF. Spectrum of clinical features in Muckle-Wells syndrome and response to anakinra. Arthritis Rheum. (2004) 50:607–12. 10.1002/art.20033 14872505

[89] Ross JB Finlayson LA Klotz PJ Langley RG Gaudet R Thompson K. Use of anakinra (Kineret) in the treatment of familial cold autoinflammatory syndrome with a 16-month follow-up. J Cutan Med Surg. (2008) 12:8–16. 10.2310/7750.2008.07050 18258152

[90] Hoffman HM Rosengren S Boyle DL Cho JY Nayar J Mueller JL. Prevention of cold-associated acute inflammation in familial cold autoinflammatory syndrome by interleukin-1 receptor antagonist. Lancet. (2004) 364:1779–85. 10.1016/S0140-6736(04)17401-1 15541451 PMC4321997

[91] Neven B Marvillet I Terrada C Ferster A Boddaert N Couloignier V. Long-term efficacy of the interleukin-1 receptor antagonist anakinra in ten patients with neonatal-onset multisystem inflammatory disease/chronic infantile neurologic, cutaneous, articular syndrome. Arthritis Rheum. (2010) 62:258–67. 10.1002/art.25057 20039428

[92] Leslie KS Lachmann HJ Bruning E McGrath JA Bybee A Gallimore JR. Phenotype, genotype, and sustained response to anakinra in 22 patients with autoinflammatory disease associated with CIAS-1/NALP3 mutations. Arch Dermatol. (2006) 142:1591–7. 10.1001/archderm.142.12.1591 17178985

[93] Cantarini L Vitale A Galeazzi M Frediani B. A case of resistant adult-onset periodic fever, aphthous stomatitis, pharyngitis and cervical adenitis (PFAPA) syndrome responsive to anakinra. Clin Exp Rheumatol. (2012) 30:593. 22931584

[94] Stojanov S Lapidus S Chitkara P Feder H Salazar JC Fleisher TA. Periodic fever, aphthous stomatitis, pharyngitis, and adenitis (PFAPA) is a disorder of innate immunity and Th1 activation responsive to IL-1 blockade. Proc Natl Acad Sci USA. (2011) 108:7148–53. 10.1073/pnas.1103681108 21478439 PMC3084055

[95] De Benedetti F Gattorno M Anton J Ben-Chetrit E Frenkel J Hoffman HM. Canakinumab for the treatment of autoinflammatory recurrent fever syndromes. N Engl J Med. (2018) 378:1908–19. 10.1056/NEJMoa1706314 29768139

[96] Brik R Butbul-Aviel Y Lubin S Ben Dayan E Rachmilewitz-Minei T Tseng L. Canakinumab for the treatment of children with colchicine-resistant familial mediterranean fever: a 6-month open-label, single-arm pilot study. Arthritis Rheumatol. (2014) 66:3241–3. 10.1002/art.38777 25049046

[97] Gul A Ozdogan H Erer B Ugurlu S Kasapcopur O Davis N. Efficacy and safety of canakinumab in adolescents and adults with colchicine-resistant familial mediterranean fever. Arthritis Res Ther. (2015) 17:243. 10.1186/s13075-015-0765-4 26337145 PMC4559892

[98] Gattorno M Obici L Cattalini M Tormey V Abrams K Davis N. Canakinumab treatment for patients with active recurrent or chronic TNF receptor-associated periodic syndrome (TRAPS): an open-label, phase II study. Ann Rheum Dis. (2017) 76:173–8. 10.1136/annrheumdis-2015-209031 27269295 PMC5264215

[99] Arostegui JI Anton J Calvo I Robles A Iglesias E Lopez-Montesinos B. Open-Label, phase ii study to assess the efficacy and safety of canakinumab treatment in active hyperimmunoglobulinemia d with periodic fever syndrome. Arthritis Rheumatol. (2017) 69:1679–88. 10.1002/art.40146 28482144

[100] Kuemmerle-Deschner JB Hachulla E Cartwright R Hawkins PN Tran TA Bader-Meunier B. Two-year results from an open-label, multicentre, phase III study evaluating the safety and efficacy of canakinumab in patients with cryopyrin-associated periodic syndrome across different severity phenotypes. Ann Rheum Dis. (2011) 70:2095–102. 10.1136/ard.2011.152728 21859692

[101] Caorsi R Lepore L Zulian F Alessio M Stabile A Insalaco A. The schedule of administration of canakinumab in cryopyrin associated periodic syndrome is driven by the phenotype severity rather than the age. Arthritis Res Ther. (2013) 15:R33. 10.1186/ar4184 23442610 PMC3672768

[102] Lachmann HJ Kone-Paut I Kuemmerle-Deschner JB Leslie KS Hachulla E Quartier P. Use of canakinumab in the cryopyrin-associated periodic syndrome. N Engl J Med. (2009) 360:2416–25. 10.1056/NEJMoa0810787 19494217

[103] Kone-Paut I Lachmann HJ Kuemmerle-Deschner JB Hachulla E Leslie KS Mouy R. Sustained remission of symptoms and improved health-related quality of life in patients with cryopyrin-associated periodic syndrome treated with canakinumab: results of a double-blind placebo-controlled randomized withdrawal study. Arthritis Res Ther. (2011) 13:R202. 10.1186/ar3535 22152723 PMC3334655

[104] Kuemmerle-Deschner JB Wittkowski H Tyrrell PN Koetter I Lohse P Ummenhofer K. Treatment of muckle-wells syndrome: analysis of two IL-1-blocking regimens. Arthritis Res Ther. (2013) 15:R64. 10.1186/ar4237 23718630 PMC4060562

[105] Soylu A Yildiz G Torun Bayram M Kavukcu S. IL-1beta blockade in periodic fever, aphthous stomatitis, pharyngitis, and cervical adenitis (PFAPA) syndrome: case-based review. Rheumatol Int. (2019). 10.1007/s00296-019-04389-3. [Epub ahead of print]. 31324971

[106] Lopalco G Rigante D Vitale A Caso F Iannone F Cantarini L. Canakinumab efficacy in refractory adult-onset PFAPA syndrome. Int J Rheum Dis. (2017) 20:1050–1. 10.1111/1756-185X.12722 26200174

[107] Hashkes PJ Spalding SJ Giannini EH Huang B Johnson A Park G Rilonacept for colchicine-resistant or -intolerant familial mediterranean fever: a randomized trial. Ann Intern Med. (2012) 157:533–41. 10.7326/0003-4819-157-8-201210160-00003 23070486

[108] Hoffman HM Throne ML Amar NJ Sebai M Kivitz AJ Kavanaugh A. Efficacy and safety of rilonacept (interleukin-1 trap) in patients with cryopyrin-associated periodic syndromes: results from two sequential placebo-controlled studies. Arthritis Rheum. (2008) 58:2443–52. 10.1002/art.23687 18668535

[109] Hoffman HM Throne ML Amar NJ Cartwright RC Kivitz AJ Soo Y. Long-term efficacy and safety profile of rilonacept in the treatment of cryopryin-associated periodic syndromes: results of a 72-week open-label extension study. Clin Ther. (2012) 34:2091–103. 10.1016/j.clinthera.2012.09.009 23031624

[110] Soriano A Verecchia E Afeltra A Landolfi R Manna R. IL-1beta biological treatment of familial Mediterranean fever. Clin Rev Allergy Immunol. (2013) 45:117–30. 10.1007/s12016-013-8358-y 23322405

[111] Urien S Bardin C Bader-Meunier B Mouy R Compeyrot-Lacassagne S Foissac F. Anakinra pharmacokinetics in children and adolescents with systemic-onset juvenile idiopathic arthritis and autoinflammatory syndromes. BMC Pharmacol Toxicol. (2013) 14:40. 10.1186/2050-6511-14-40 23915458 PMC3750485

[112] Kim DC Reitz B Carmichael DF Bloedow DC. Kidney as a major clearance organ for recombinant human interleukin-1 receptor antagonist. J Pharm Sci. (1995) 84:575–80. 10.1002/jps.2600840511 7658347

[113] Yang BB Baughman S Sullivan JT. Pharmacokinetics of anakinra in subjects with different levels of renal function. Clin Pharmacol Ther. (2003) 74:85–94. 10.1016/S0009-9236(03)00094-8 12844139

[114] Goshen I Kreisel T Ounallah-Saad H Renbaum P Zalzstein Y Ben-Hur T. A dual role for interleukin-1 in hippocampal-dependent memory processes. Psychoneuroendocrinology. (2007) 32:1106–15. 10.1016/j.psyneuen.2007.09.004 17976923

[115] Bilgen SA Kilic L Akdogan A Kiraz S Kalyoncu U Karadag O Effects of anti-tumor necrosis factor agents for familial mediterranean fever patients with chronic arthritis and/or sacroiliitis who were resistant to colchicine treatment. J Clin Rheumatol. (2011) 17:358–62. 10.1097/RHU.0b013e31823682f5 21946459

[116] Ozgocmen S Akgul O. Anti-TNF agents in familial mediterranean fever: report of three cases and review of the literature. Mod Rheumatol. (2011) 21:684–90. 10.3109/s10165-011-0463-2 21567247

[117] Ozen S Kuemmerle-Deschner JB Cimaz R Livneh A Quartier P Kone-Paut I. International retrospective chart review of treatment patterns in severe FMF, TRAPS and MKD/HIDS. Arthritis Care Res. (2017) 69:578–86. 10.1002/acr.23120 27723279

[118] Bulua AC Mogul DB Aksentijevich I Singh H He DY Muenz LR. Efficacy of etanercept in the tumor necrosis factor receptor-associated periodic syndrome: a prospective, open-label, dose-escalation study. Arthritis Rheum. (2012) 64:908–13. 10.1002/art.33416 22006113 PMC3882089

[119] Matsubara T Hasegawa M Shiraishi M Hoffman HM Ichiyama T Tanaka T. A severe case of chronic infantile neurologic, cutaneous, articular syndrome treated with biologic agents. Arthritis Rheum. (2006) 54:2314–20. 10.1002/art.21965 16802372

[120] Fujikawa K Migita K Tsukada T Umeda M Nonaka F Kawakami A. Interleukin-6 targeting therapy in familial mediterranean fever. Clin Exp Rheumatol. (2013) 31:150–1. 24064027

[121] Hamanoue S Suwabe T Hoshino J Sumida K Mise K Hayami N Successful treatment with humanized anti-interleukin-6 receptor antibody (tocilizumab) in a case of AA amyloidosis complicated by familial mediterranean fever. Mod Rheumatol. (2016) 26:610–3. 10.3109/14397595.2014.908810 25619282

[122] Serelis J Christaki S Skopouli FN. Remission of nephrotic syndrome due to AA-amyloidosis, complicating familiar mediterranean fever, with tocilizumab. Clin Exp Rheumatol. (2015) 33:S169. 25572486

[123] Umeda M Aramaki T Fujikawa K Iwamoto N Ichinose K Terada K. Tocilizumab is effective in a familial mediterranean fever patient complicated with histologically proven recurrent fasciitis and myositis. Int J Rheum Dis. (2017) 20:1868–71. 10.1111/1756-185X.12776 26481326

[124] Nikiphorou E Neocleous V Phylactou LA Psarelis S. Successful use of tocilizumab in two cases of severe autoinflammatory disease with a single copy of the mediterranean fever gene. Rheumatology. (2017) 56:1627–8. 10.1093/rheumatology/kex180 28486679

[125] Aikawa E Shimizu T Koga T Endo Y Umeda M Hori T. Atypical familial mediterranean fever complicated with gastrointestinal amyloidosis diagnosed due to paroxysmal arthralgia and intractable diarrhea, successfully treated with tocilizumab. Intern Med. (2019) 58:1781–5. 10.2169/internalmedicine.2277-18 30713308 PMC6630114

[126] Yilmaz S Cinar M Simsek I Erdem H Pay S. Tocilizumab in the treatment of patients with AA amyloidosis secondary to familial mediterranean fever. Rheumatology. (2015) 54:564–5. 10.1093/rheumatology/keu474 25504961

[127] Ugurlu S Hacioglu A Adibnia Y Hamuryudan V Ozdogan H. Tocilizumab in the treatment of twelve cases with aa amyloidosis secondary to familial mediterranean fever. Orphanet J Rare Dis. (2017) 12:105. 10.1186/s13023-017-0642-0 28558744 PMC5450086

[128] Yilmaz S Tekgoz E Cinar M. Recurrence of proteinuria after cessation of tocilizumab in patients with AA amyloidosis secondary to FMF. Eur J Rheumatol. (2018) 5:278–80. 10.5152/eurjrheum.2018.17183 30071938 PMC6267750

[129] La Torre F Muratore M Vitale A Moramarco F Quarta L Cantarini L. Canakinumab efficacy and long-term tocilizumab administration in tumor necrosis factor receptor-associated periodic syndrome (TRAPS). Rheumatol Int. (2015) 35:1943–7. 10.1007/s00296-015-3305-2 26048626

[130] Vaitla PM Radford PM Tighe PJ Powell RJ McDermott EM Todd I Role of interleukin-6 in a patient with tumor necrosis factor receptor-associated periodic syndrome: assessment of outcomes following treatment with the anti-interleukin-6 receptor monoclonal antibody tocilizumab. Arthritis Rheum. (2011) 63:1151–5. 10.1002/art.30215 21225679

[131] Akasbi N Soyfoo MS. Successful treatment of tumor necrosis factor receptor-associated periodic syndrome (TRAPS) with tocilizumab: a case report. Eur J Rheumatol. (2015) 2:35–6. 10.5152/eurjrheumatol.2014.14053 27708919 PMC5047242

[132] Shendi HM Devlin LA Edgar JD. Interleukin 6 blockade for hyperimmunoglobulin D and periodic fever syndrome. J Clin Rheumatol. (2014) 20:103–5. 10.1097/01.RHU.0000442576.41537.de 24561416

[133] Stoffels M Jongekrijg J Remijn T Kok N van der Meer JW Simon A. TLR2/TLR4-dependent exaggerated cytokine production in hyperimmunoglobulinaemia D and periodic fever syndrome. Rheumatology. (2015) 54:363–8. 10.1093/rheumatology/keu341 25173351

[134] Lane T Gillmore JD Wechalekar AD Hawkins PN Lachmann HJ. Therapeutic blockade of interleukin-6 by tocilizumab in the management of AA amyloidosis and chronic inflammatory disorders: a case series and review of the literature. Clin Exp Rheumatol. (2015) 33:S46–53. 26120866

[135] Musters A Tak PP Baeten DL Tas SW. Anti-interleukin 6 receptor therapy for hyper-IgD syndrome. BMJ Case Rep. (2015) 2015:bcr2015210513. 10.1136/bcr-2015-210513 26516243 PMC4636692

[136] Rafiq NK Lachmann H Joensen F Herlin T Brogan PA. Tocilizumab for the treatment of mevalonate kinase deficiency. Case Rep Pediatr. (2018) 2018:3514645. 10.1155/2018/3514645 30225156 PMC6129367

[137] Snegireva LS Kostik MM Caroli F Ceccherini I Gattorno M Chasnyk VG. Failure of tocilizumab treatment in a CINCA patient: clinical and pathogenic implications. Rheumatology. (2013) 52:1731–2. 10.1093/rheumatology/ket121 23481539

[138] Gok K Cengiz G Erol K Ozgocmen S. Tofacitinib suppresses disease activity and febrile attacks in a patient with coexisting rheumatoid arthritis and familial mediterranean fever. Acta Reumatol Port. (2017) 42:88–90. 28371574

[139] Garcia-Robledo JE Aragon CC Nieto-Aristizabal I Posso-Osorio I Canas CA Tobon GJ Tofacitinib for familial mediterranean fever: a new alternative therapy? Rheumatology. (2019) 58:553–4. 10.1093/rheumatology/key384 30535114

[140] Karadeniz H Guler AA Atas N Satis H Salman RB Babaoglu H. Tofacitinib for the treatment for colchicine-resistant familial mediterranean fever: case-based review. Rheumatol Int. (2019) 40:169–73. 10.1007/s00296-019-04490-7 31813060

[141] Fleischmann RM Schechtman J Bennett R Handel ML Burmester GR Tesser J. Anakinra, a recombinant human interleukin-1 receptor antagonist (r-metHuIL-1ra), in patients with rheumatoid arthritis: a large, international, multicenter, placebo-controlled trial. Arthritis Rheum. (2003) 48:927–34. 10.1002/art.10870 12687534

[142] Fleischmann RM Tesser J Schiff MH Schechtman J Burmester GR Bennett R. Safety of extended treatment with anakinra in patients with rheumatoid arthritis. Ann Rheum Dis. (2006) 65:1006–12. 10.1136/ard.2005.048371 16396977 PMC1798263

[143] Galloway JB Hyrich KL Mercer LK Dixon WG Watson KD Lunt M. The risk of serious infections in patients receiving anakinra for rheumatoid arthritis: results from the british society for rheumatology biologics register. Rheumatology. (2011) 50:1341–2. 10.1093/rheumatology/ker146 21489973

[144] Kafka D Ling E Feldman G Benharroch D Voronov E Givon-Lavi N. Contribution of IL-1 to resistance to Streptococcus pneumoniae infection. Int Immunol. (2008) 20:1139–46. 10.1093/intimm/dxn071 18596024

[145] Mertens M Singh JA Anakinra for rheumatoid arthritis. Cochrane Database Syst Rev. (2009) CD005121. 10.1002/14651858.CD005121.pub3 PMC12296252 19160248

[146] Gotestam Skorpen C Hoeltzenbein M Tincani A Fischer-Betz R Elefant E Chambers C. The EULAR points to consider for use of antirheumatic drugs before pregnancy, and during pregnancy and lactation. Ann Rheum Dis. (2016) 75:795–810. 10.1136/annrheumdis-2015-208840 26888948

[147] Venhoff N Voll RE Glaser C Thiel J. [IL-1-blockade with anakinra during pregnancy : retrospective analysis of efficacy and safety in female patients with familial mediterranean fever]. Z Rheumatol. (2018) 77:127–34. 10.1007/s00393-017-0354-9 28752409

[148] Chang Z Spong C Jesus AA Davis M Plass N Stone DL. Anakinra use during pregnancy in patients with cryopyrin-associated periodic syndromes (CAPS). Arthritis Rheumatol. (2014) 66:3227–32. 10.1002/art.38811 25223501 PMC4323990

[149] Rodriguez-Smith J Lin YC Tsai WL Kim H Montealegre-Sanchez G Chapelle D. Cerebrospinal fluid cytokines correlate with aseptic meningitis and blood-brain barrier function in neonatal-onset multisystem inflammatory disease: central nervous system biomarkers in neonatal-onset multisystem inflammatory disease correlate with central nervous system inflammation. Arthritis Rheumatol. (2017) 69:1325–36. 10.1002/art.40055 28118536 PMC5449229

[150] Sibley CH Chioato A Felix S Colin L Chakraborty A Plass N. A 24-month open-label study of canakinumab in neonatal-onset multisystem inflammatory disease. Ann Rheum Dis. (2015) 74:1714–9. 10.1136/annrheumdis-2013-204877 24906637 PMC4258169

[151] Chakraborty A Tannenbaum S Rordorf C Lowe PJ Floch D Gram H. Pharmacokinetic and pharmacodynamic properties of canakinumab, a human anti-interleukin-1beta monoclonal antibody. Clin Pharmacokinet. (2012) 51:e1–18. 10.2165/11599820-000000000-00000 22550964 PMC3584253

[152] Youngstein T Hoffmann P Gul A Lane T Williams R Rowczenio DM. International multi-centre study of pregnancy outcomes with interleukin-1 inhibitors. Rheumatology. (2017) 56:2102–8. 10.1093/rheumatology/kex305 28968868 PMC6251516

[153] Brogan PHM Kuemmerle-Deschner J Efficacy and safety of canakinumab in patients with cryopyrin associated periodic syndromes: an open- label, phase-III, extension study. Ann Rheum Dis. (2016) 75(Suppl. 2):620–1. 10.1136/annrheumdis-2016-eular.4865 21859692

[154] Ridker PM Everett BM Thuren T MacFadyen JG Chang WH Ballantyne C. Antiinflammatory therapy with canakinumab for atherosclerotic disease. N Engl J Med. (2017) 377:1119–31. 10.1056/NEJMoa1707914 28845751

[155] Ilowite NT Prather K Lokhnygina Y Schanberg LE Elder M Milojevic D. Randomized, double-blind, placebo-controlled trial of the efficacy and safety of rilonacept in the treatment of systemic juvenile idiopathic arthritis. Arthritis Rheumatol. (2014) 66:2570–9. 10.1002/art.38699 24839206 PMC4314719

[156] Autmizguine J Cohen-Wolkowiez M Ilowite N Investigators R. Rilonacept pharmacokinetics in children with systemic juvenile idiopathic arthritis. J Clin Pharmacol. (2015) 55:39–44. 10.1002/jcph.372 25079592 PMC4276471

[157] Radin A Marbury T Osgood G Belomestnov P. Safety and pharmacokinetics of subcutaneously administered rilonacept in patients with well-controlled end-stage renal disease (ESRD). J Clin Pharmacol. (2010) 50:835–41. 10.1177/0091270009351882 20035038

[158] Sundy JS Schumacher HR Kivitz A Weinstein SP Wu R King-Davis S. Rilonacept for gout flare prevention in patients receiving uric acid-lowering therapy: results of RESURGE, a phase III, international safety study. J Rheumatol. (2014) 41:1703–11. 10.3899/jrheum.131226 25028379

[159] van der Hilst J Moutschen M Messiaen PE Lauwerys BR Vanderschueren S. Efficacy of anti-IL-1 treatment in familial mediterranean fever: a systematic review of the literature. Biologics. (2016) 10:75–80. 10.2147/BTT.S102954 27110096 PMC4831592

[160] Babaoglu H Varan O Kucuk H Atas N Satis H Salman R. On demand use of anakinra for attacks of familial mediterranean fever (FMF). Clin Rheumatol. (2019) 38:577–81. 10.1007/s10067-018-4230-z 30062447

[161] Cetin P Sari I Sozeri B Cam O Birlik M Akkoc N. Efficacy of interleukin-1 targeting treatments in patients with familial mediterranean fever. Inflammation. (2015) 38:27–31. 10.1007/s10753-014-0004-1 25139580

[162] Eroglu FK Besbas N Topaloglu R Ozen S. Treatment of colchicine-resistant familial mediterranean fever in children and adolescents. Rheumatol Int. (2015) 35:1733–7. 10.1007/s00296-015-3293-2 26001859

[163] Mitroulis I Skendros P Oikonomou A Tzioufas AG Ritis K. The efficacy of canakinumab in the treatment of a patient with familial mediterranean fever and longstanding destructive arthritis. Ann Rheum Dis. (2011) 70:1347–8. 10.1136/ard.2010.146878 21345814

[164] Kone-Paut I Piram M Benseler S Hofer M Lachmann H Hoffman HM Improvement of disease activity in patients with colchicine-resistant FMF, HIDS/MKD and TRAPS assessed by autoinflammatory disease activity index (AIDAI): results from the cluster trial. Ann Rheum Dis. (2017) 76 (Suppl. 2):398–9. 10.1136/annrheumdis-2017-eular.5655

[165] Grimwood C Despert V Jeru I Hentgen V. On-demand treatment with anakinra: a treatment option for selected TRAPS patients. Rheumatology. (2015) 54:1749–51. 10.1093/rheumatology/kev111 26078218

[166] Almeida MQ Tsang KM Cheadle C Watkins T Grivel JC Nesterova M. Protein kinase a regulates caspase-1 via Ets-1 in bone stromal cell-derived lesions: a link between cyclic AMP and pro-inflammatory pathways in osteoblast progenitors. Hum Mol Genet. (2011) 20:165–75. 10.1093/hmg/ddq455 20940146 PMC3000682

[167] Yokoyama K Ikeya M Umeda K Oda H Nodomi S Nasu A. Enhanced chondrogenesis of induced pluripotent stem cells from patients with neonatal-onset multisystem inflammatory disease occurs via the caspase 1-independent cAMP/protein kinase A/CREB pathway. Arthritis Rheumatol. (2015) 67:302–14. 10.1002/art.38912 25302486

[168] Galea J Ogungbenro K Hulme S Greenhalgh A Aarons L Scarth S. Intravenous anakinra can achieve experimentally effective concentrations in the central nervous system within a therapeutic time window: results of a dose-ranging study. J Cereb Blood Flow Metab. (2011) 31:439–47. 10.1038/jcbfm.2010.103 20628399 PMC3049499

[169] Ahmadi N Brewer CC Zalewski C King KA Butman JA Plass N. Cryopyrin-associated periodic syndromes: otolaryngologic and audiologic manifestations. Otolaryngol Head Neck Surg. (2011) 145:295–302. 10.1177/0194599811402296 21493283 PMC3407887

[170] Kullenberg T Lofqvist M Leinonen M Goldbach-Mansky R Olivecrona H. Long-term safety profile of anakinra in patients with severe cryopyrin-associated periodic syndromes. Rheumatology. (2016) 55:1499–506. 10.1093/rheumatology/kew208 27143789 PMC4957676

[171] Nedjai B Quillinan N Coughlan RJ Church L McDermott MF Hitman GA. Lessons from anti-TNF biologics: infliximab failure in a TRAPS family with the T50M mutation in TNFRSF1A. Adv Exp Med Biol. (2011) 691:409–19. 10.1007/978-1-4419-6612-4_43 21153346

[172] Sanchez GAM Reinhardt A Ramsey S Wittkowski H Hashkes PJ Berkun Y. JAK1/2 inhibition with baricitinib in the treatment of autoinflammatory interferonopathies. J Clin Invest. (2018) 128:3041–52. 10.1172/JCI98814 29649002 PMC6026004

[173] Wenzel J van Holt N Maier J Vonnahme M Bieber T Wolf D. JAK1/2 Inhibitor ruxolitinib controls a case of chilblain lupus erythematosus. J Invest Dermatol. (2016) 136:1281–3. 10.1016/j.jid.2016.02.015 26916391

[174] Konig N Fiehn C Wolf C Schuster M Cura Costa E Tungler V. Familial chilblain lupus due to a gain-of-function mutation in STING. Ann Rheum Dis. (2017) 76:468–72. 10.1136/annrheumdis-2016-209841 27566796

[175] Crow YJ Vanderver A Orcesi S Kuijpers TW Rice GI. Therapies in aicardi-goutieres syndrome. Clin Exp Immunol. (2014) 175:1–8. 10.1111/cei.12115 23607857 PMC3898548

[176] Zahid A Li B Kombe AJK Jin T Tao J. Pharmacological inhibitors of the NLRP3 inflammasome. Front Immunol. (2019) 10:2538. 10.3389/fimmu.2019.02538 31749805 PMC6842943

[177] Koga T Sato S Miyamoto J Hagimori N Kawazoe Y Arinaga K Comparison of the efficacy and safety of tocilizumab forcolchicine-resistant or colchicine-intolerant familial mediterranean fever: study protocol for an investigator-initiated, multicenter, randomized, double-blind, placebo-controlled trial. Trials. (2018) 19:715 10.1186/s13063-018-3105-6 30594222 PMC6311086

[178] Koga T Hagimori N Sato S Morimoto S Hosogaya N Fukushima C. An open-label continuation trial of tocilizumab for familial mediterranean fever with colchicine ineffective or intolerance: Study protocol for investigator-initiated, multicenter, open-label trial. Medicine. (2020) 99:e18328. 10.1097/MD.0000000000018328 31895769 PMC6946311

